# Current Molecular Biology and Therapeutic Strategy Status and Prospects for circRNAs in HBV-Associated Hepatocellular Carcinoma

**DOI:** 10.3389/fonc.2021.697747

**Published:** 2021-07-02

**Authors:** Rui Liao, Lei Liu, Jian Zhou, Xufu Wei, Ping Huang

**Affiliations:** ^1^ Department of Hepatobiliary Surgery, The First Affiliated Hospital of Chongqing Medical University, Chongqing, China; ^2^ Department of Hepatobiliary Surgery, The People’s Rongchang Hospital, Chongqing, China

**Keywords:** circular RNA, microRNA, hepatitis B, hepatocellular carcinoma, biomarker, drug resistance

## Abstract

Circular RNAs (circRNAs) are newly classified noncoding RNA (ncRNA) members with a covalently closed continuous loop structure that are involved in immune responses against hepatitis B virus (HBV) infections and play important biological roles in the occurrence and pathogenesis of HCC progression. The roles of circRNAs in HBV-associated HCC (HBV-HCC) have gained increasing attention. Substantial evidence has revealed that both tissue and circulating circRNAs may serve as potential biomarkers for diagnostic, prognostic and therapeutic purposes. So far, at least four circRNA/miRNA regulatory axes such as circRNA_101764/miR-181, circRNA_100338/miR-141-3p, circ-ARL3/miR-1305, circ-ATP5H/miR-138-5p, and several circulating circRNAs were reported to be associated with HBV-HCC development. Notably, TGF/SMAD, JAK/STAT, Notch and Wnt/β-catenin signaling pathways may play pivotal roles in this HBV-driven HCC *via* several circRNAs. Moreover, in non-HBV HCC patients or HCC patients partially infected by HBV, numerous circRNAs have been identified to be important regulators impacting the malignant biological behavior of HCC. Furthermore, the role of circRNAs in HCC drug resistance has become a focus of research with the aim of reversing chemoresistance and immune resistance. Herein, we review the molecular biology of circRNAs in HBV-HCC and their potential in therapeutic strategies.

## Introduction

Hepatocellular carcinoma (HCC), mainly induced by hepatitis B (HBV) or C viral (HCV) infection and accounting for the bulk of primary liver cancers, ranks as the fourth most common cause of cancer-related death globally in 2018 and has a notably poor prognosis (1). Unfortunately, most HCC patients are diagnosed at advanced disease stages and miss the opportunity for curative resection. Although some locoregional therapy approaches (e.g., radiofrequency ablation, RFA; transcatheter arterial chemoembolization, TACE; transcatheter arterial infusion, TAI), several approved systemic therapies (such as sorafenib, lenvatinib, and cabozantinib), and immunotherapy can partially improve the outcomes of these patients, the long-term outcomes are still generally poor (2, 3). Therefore, exploring the molecular biology of valuable biomarkers for early diagnosis of HCC and therapeutic strategies against HCC is extremely important. Circular RNAs (circRNAs) are newly classified noncoding RNA (ncRNA) members that form a covalently closed continuous loop structure and are more stable than linear mRNAs (4). Many studies have indicated that host circRNAs are involved in immune responses against HBV infection. To date, dozens of circRNAs have been reported to play important biological roles in the occurrence and pathogenesis of HCC progression (5), and they are closely related to immune responses against HBV infection and regulation of HCC tumorigenesis, including self-sustenance in growth signals, cell proliferation, angiogenesis, cell apoptosis, and tumor metastasis. In this review, we discuss the molecular biology underlying HBV-associated HCC (HBV-HCC) and thereby provide insight into the role of circRNAs in therapeutic strategies.

## General Features of circRNAs

Unlike conventional linear splicing of RNAs, circRNAs are generated from back-splicing of exons, introns, or both, which prevents them from being degraded by RNA exonucleases or RNase R. Back-splicing in circRNA synthesis occurs both cotranscriptionally and posttranscriptionally and is favored by a high rate of transcription elongation (4). Additionally, alternative back-splicing events, in particular N6-methyladenosine (m6A) modification, can occur and produce multiple circRNA isoforms (6). Hence, circRNAs have a longer half-life and more inherent stability than linear mRNAs. There are three types of circRNAs: circular exonic circRNAs (EcircRNAs), circular intronic RNAs (ciRNAs) and exon-intron circRNAs (EIciRNAs) (6). EcircRNAs are abundant in the cytoplasm, constitute the majority of circRNAs and serve as miRNA sponges. However, ciRNAs and EIciRNAs are predominantly nuclear and may modulate gene transcription and posttranscription modification (7). Exosomal circRNAs has been recognized as a potentially effective way to clear or degrade circRNAs (8). circRNAs have been found to be involved in various biological functions, including microRNA (miRNA) and protein sponging, transcriptional and protein regulation, and alternative splicing modulation, and can act as protein translation templates (7). Moreover, many studies have revealed that circRNAs can contribute to cell growth, angiogenesis, unlimited replicative potential, and cancer invasion and metastasis by acting as different miRNA sequesters or sponges and directly targeting protein-coding genes (7).

## Molecular Biology Relationship Between circRNAs and hepatitis B Virus

At present, it is impossible to completely eliminate HBV infection in the human body due to the persistence of covalently closed circular DNA (cccDNA) in the nuclei of infected hepatocytes (9). Recent evidence has shown that viruses can encode a repertoire of circRNAs (10). In accordance with the pivotal roles in the biogenesis and functions of circRNAs during virus infection, the novel mechanisms underlying the pathogenesis and progression of chronic hepatitis B (CHB) involving circRNAs are slowly being validated. To identify hepatic circRNAs associated with chronic hepatitis B (CHB), Zhou et al. performed RNA sequencing using liver biopsies from untreated CHB patients and found that a total of 99 dysregulated circRNAs were correlated with CHB. CHB-related circRNA-miRNA-mRNA pathway analysis hinted that hsa_circ_0000650 regulated transforming growth factor-β2 (TGFβ2) by sponging miR-6873-3p (11). Moreover, circRNAs regulate HBV replication by mediating host-virus interactions. It was found that viral-derived circRNAs are produced during HBV replication and are regulated by the host DHX9 (DEAH-box helicase 9) protein, which did not affect the levels of HBV DNA. Therefore, in HBV infection, the RNA binding factor DHX9 may function as a crucial regulator of viral-derived circRNAs or viral proteins (12). Furthermore, circRNAs induce an antiviral immune response. An *in vitro* study (13) revealed a high hsa_circ_0004812 expression level in CHB patients and HBV-infected hepatoma cells. The knockdown of hsa_circ_0004812 promoted IFN-α/β expression to inhibit viral replication. The overexpression of hsa_circ_0004812 stimulated HBV-induced immunosuppression through the circ_0004812/miR-1287-5p/Follistatin-related protein (FSTL) 1 axis, which promoted FSTL1 expression by inhibiting miR-1287-5p. However, due to our presently poor understanding of their expression, regulation and biological function, further investigations are needed to determine the potential mechanisms behind the different circRNA regulation patterns associated with HBV infection.

## Roles of circRNAs in HBV-HCC

**Figure 1:**
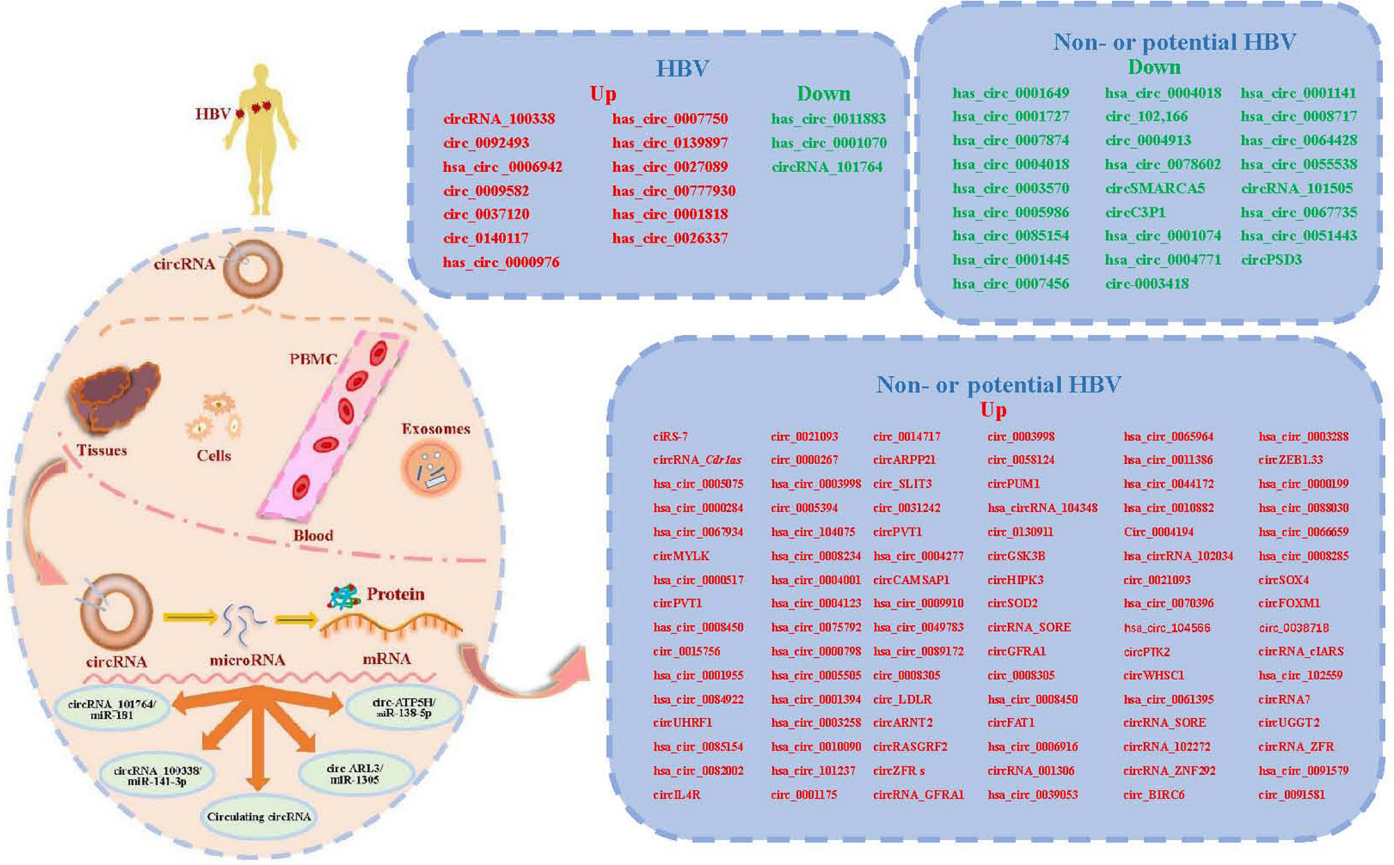
A summary diagram of circular RNAs (circRNAs) involved in circRNA-microRNA (miRNAs)-mRNA axis in hepatocellular carcinoma (HCC) with or without hepatitis B virus (HBV). CircRNAs can be found in liver tissues, cells, serum, plasma, peripheral blood mononuclear cells (PMBC) and exosomes. Most circRNAs can act as miRNA sponges or sequesters. CircRNAs may function as sponges or decoys for proteins and thereby regulate their activity. At least four reported circRNA/miRNA regulatory axes and several circulating circRNAs might contribute to the development of chronic hepatitis B (CHB) related HCC. We list the validated circRNAs on the right of this figure.

Among circRNA/miRNA interaction networks, at least five circRNA/miRNA regulatory axes might contribute to CHB development, including hepatitis B, inflammatory mediator regulation of transient receptor potential (TRP) channels, T cell receptor, TGF-β and MAPK signaling pathways (14). Obviously, these signaling pathways are closely involved in the development of HCC, including cell proliferation, apoptosis, migration, and invasion and so on. However, there are a few mechanistic studies, especially in HBV-HCC, on other circRNA/miRNA regulatory axes. Therefore, the information about the detailed mechanisms of circRNA/miRNA regulatory axes is limit. Here, we systematically summarize the literature on other validated circRNA/miRNA/target gene axes associated with HBV-HCC (**Figure 1** and **Table 1**).

**Table 1:** Overview of the identified circular RNA in HBV associated hepatocellular carcinoma.

circRNA	Gene symbol	miR Target	Target genes/proteins	Sample	Function	Ref
**Up-regulation**						
circRNA_100338	SNX27	miR-141-3p	MTSS1	Tissues, cell line	Not investigated	(15)
circ_0092493	ARL3	miR-1305	WNT2, UBE2T, MDM2, TGF-β2, POLR3G	Tissues, cell line	Promotes cell proliferation and invasion	(16)
has_circ _0006942	ATP5H	miR-138-5p	TNFAIP3	Tissues, cell line	Promotes HBV replication and expression	(17)
circ_0009582	RERE	Not investigated	Not investigated	Plasma	Not investigated	(18)
circ_0037120	RHBDF1	Not investigated	Not investigated	Plasma	Not investigated	(18)
circ_0140117	CNKSR2	Not investigated	Not investigated	Plasma	Not investigated	(18)
has_circ_0000976	HPCAL1	Not investigated	Not investigated	Plasma, tissues, cell line	Not investigated	(19)
has_circ_0007750	RABGGTA	Not investigated	Not investigated	Plasma, tissues, cell line	Not investigated	(19)
has_circ_0139897	MTM1	Not investigated	Not investigated	Plasma, tissues, cell line	Not investigated	(19)
has_circ_0027089	PTGES3	Not investigated	Not investigated	Plasma	Not investigated	(20)
has_circ_00777930	AHI1	Not investigated	Not investigated	Plasma	Not investigated	(20)
has_circ_0001818	UBR5	Not investigated	Not investigated	Plasma	Not investigated	(20)
has_circ_0026337	SCN8A	Not investigated	Not investigated	Plasma	Not investigated	(20)
**Down-regulation**						
has_circ_0011883	PPT1	Not investigated	Not investigated	Plasma	Not investigated	(20)
has_circ_0001070	R3HDM1	Not investigate	Not investigated	Plasma	Not investigated	(20)
circRNA_101764	MIPOL1	miR-181	PI3K-Akt	Tissues	Not investigated	(21)

### circRNA_101764/miR-181

circRNA/miRNA interaction networks were constructed to predict the function of these circRNAs in CHB. Increased evidence from circRNA microarrays has confirmed that circRNA-miRNA-mRNA networks based on specific functional circRNAs may facilitate hepatocarcinogenesis in HBV-HCC. For example, bioinformatics analyses of a circRNA microarray from three HCC and paired adjacent nontumorous tissues indicated 24 upregulated and 23 downregulated differentially expressed circRNAs (HCC *vs* nontumors, fold change>2.0 and P<0.05) (21). Then, 3 upregulated (hsa_circRNA_102814, 100381, and 103489) and 3 downregulated (hsa_circRNA_101764, 100327, and 103361) miRNAs were verified by qRT-PCR. Of them, hsa_circRNA_101764, coexpressed with the miR-181 family, was the largest node in the circRNA/microRNA coexpression network (21). By activating epigenetic upregulation of miR-181, HBV-encoded X antigen (HBx) could promote “stemness” in the pathogenesis of HCC (22). GO analysis of this circRNA microarray revealed that genes in the PI3K-Akt signaling pathway were the most abundant target genes involved in circRNA/miRNA interactions (21). The PI3K-Akt signaling pathway has already been verified to include oncogenes that functionally contribute to hepatocarcinogenesis by inducing malignant transformation of hepatocytes (23). Hence, circRNA_101764/miR-181/PI3K may play an important role in the cell network during HBV-HCC hepatocarcinogenesis.

### circRNA_100338/miR-141-3p

Another circRNA microarray of HBV-HCC performed by Huang et al. (15) identified a total of 189 significantly upregulated and 37 downregulated circRNAs. Of note, circRNA_100338, which is significantly more highly expressed in HCC tissue than in paired pericancerous tissue, is closely correlated with HBV-HCC metastatic progression and consequently the cumulative survival rate. In silico and experimental analyses suggested that miR-141-3p is a direct target of circRNA_100338 to regulate the gene expression necessary for HCC carcinogenesis (15). On the other hand, this study also found that metastasis suppressor 1 (MTSS1) is very likely a potential target of miR-141-3p, which may act as an oncogene and a driver of metastasis in HBV-HCC through a potential circRNA_100338-miR141-3p-MTSS1 interaction pathway. As a tumor inhibitor in HCC, miR 141 can suppress HCC cell growth, invasion and metastasis by directly targeting TGFβR1 (24), sperm-associated antigen 9 (25), hepatocyte nuclear factor-3β (26), T lymphoma invasion and metastasis 1 (27) and their downstream signaling cascade. In a study employing an orthotopic nude mouse model and cell lines (28), downregulation of MTSS1 decreased the invasion potential of HBV-HCC *in vitro* and averted the extent of lung metastasis *in vivo*. Based on these findings, circRNA_100338/miR-141-3p/MTSS1 could be used as a prediction biomarker for HBV-HCC patient outcomes and as a potential therapeutic target.

### circ-ARL3/miR-1305

circ-ARL3, also known as hsa_circ_0092493, was reported to be significantly upregulated in HBV-positive HCC cells and tissues (16). A circRNA expression profile in HBV^+^ HepG2.2.15 cells and their parental HBV^−^ HepG2 cells found 22 upregulated and 63 downregulated circRNAs. Among them, circ-ARL3 had the greatest differential expression, which was positively associated with positive HBsAg test results, larger tumor size and advanced clinical stage. The upregulation of circ-ARL3 is attributed to N6-methyladenosine (m6A) modification induced by HBx protein (16). Importantly, circ-ARL3 serves as a molecular sponge of miR-1305, antagonizing the inhibitory effects of miR-1305 in a cohort of target oncogenes (16), including WNT2 (29), ubiquitin-conjugating enzyme E2 T (UBE2T) (30), double minute 2 homolog (MDM2) (31), transforming growth factor-beta2 (TGF-β2) (32), and RNA Polymerase III Subunit G (POLR3G) (33), thereby facilitating HBV-HCC progression. Wei et al. demonstrated that miR-1305 targeted ubiquitin-conjugating enzyme E2T (UBE2T) to suppress the Akt signaling pathway and then prevented the self-renewal and tumorigenicity of cancer stem cells in HCC (30). Therefore, circ-ARL3/miR-1305 is a critical carcinogenic signaling pathway involved in the primary pathogenesis of HBV-HCC.

### circ-ATP5H/miR-138-5p

circ-ATP5H, also known as hsa_circ_0006942, is expressed at high levels in HBV+ HCC tissues and cells (17). circ-ATP5H knockdown prevented HBV DNA replication and hindered HBsAg and HBeAg expression in HBV-positive cells. Moreover, circ-ATP5H sponges miR-138-5p to upregulate tumor necrosis factor alpha-induced protein 3 (TNFAIP3) (17). Several recent studies have revealed that miR-138-5p plays a pivotal regulatory role in HCC by mediating a series of biological processes, including chemoresistance, cell proliferation, cell migration, invasion, metastasis and tumorigenesis (34, 35). Furthermore, TNFAIP3 has already been identified as an important regulator of HBV DNA replication and of cell proliferation and apoptosis in HBV-HCC (36). Thus, circ-ATP5H may play an important role in HBV-HCC development and progression by modulating the miR-138-5p/TNFAIP3 axis.

### Circulating circRNA

In addition to circRNAs expressed in tissues and cells, some circulating circRNAs have been demonstrated to be involved in HBV-HCC occurrence. In a microarray-based high-throughput screening of HBV-HCC-related circulating circRNAs, stratified risk score analysis verified that circ_0009582, circ_0037120 and circ_0140117 were candidate circulating fingerprints for distinguishing HCC patients from those with chronic hepatitis and healthy people (18). Zhu et al. investigated plasma circRNAs in 10 HBV-HCC patients and 5 HBV-related liver cirrhosis patients using a microarray to screen differentially expressed circRNAs (20). A total of 157 upregulated and 161 downregulated circRNAs were found. Of them, hsa_circ_0027089 exhibited the highest significance and further distinguished HCC patients from cirrhosis patients and healthy participants. A large-scale, multicenter study also employed a microarray and qPCR to explore plasma circRNAs increased in HBV HCC patients (19). They identified a plasma circRNA panel (CircPanel) containing three circRNAs (hsa_circ_0000976, hsa_circ_0007750 and hsa_circ_0139897) that could detect HBV-HCC. Although there have been few in-depth mechanistic studies of circulating circRNAs, these findings provide evidence that these circRNAs might participate in HBV-HCC progression.

### circRNAs in Non-HBV and Potential HBV-HCC

In this review, we have mainly focused on the roles of circRNAs in HBV-HCC. However, to date, in non-HBV HCC patients or HCC patients partially infected by HBV, numerous circRNAs have been reported to be important regulators impacting the malignant biological behavior of tumors. Additionally, in some research, although HBV-HCC was not the focus of the studies, the majority of the HCC population had HBV infection. We also found that the expression and roles of some circRNAs in HCC have been inconsistently reported, such as circRNA-103809 (37–39). Functionally, circRNA activations are closely associated with cancer cell proliferation, cycle progression, cell apoptosis, migration, invasion and Epithelial-mesenchymal transition (EMT) during HCC process. Herein, we also summarize the relationship between circRNAs and non-HBV or potential HBV-HCC from biological function to clinical significance. Whether these circRNAs can be biomarkers for the diagnosis of HBV-HCC patients and prognosis determination should be evaluated in the future (**Table 2**).

**Table 2:** Summary of circular RNA in non- and potential HBV infected hepatocellular carcinoma population.

circRNA	Gene symbol	miR Target	Target genes/proteins	HBV infection	Function	Ref
**Up-regulation**						
ciRS-7 circRNA_Cdr1as	CDR1AS	miR-7	PIK3CD, p70S6K, mTOR, CCNE1, PIK3CD	104/108HBV-HCC patients	Promotes cell proliferation and invasion	(40, 41)
has_circ_0005075	EIF4 gG3	miR-23b-5p, miR-93-3p, miR-581, miR-23a-5p	GO and KEGG pathway analysis	23/30 HBV-HCC patients	Promotes cell adhesion	(42)
has_circ_0000284	HIPK3	miR-124	AQP3	41/50 HBV-HCC patients	Promotes cell proliferation and migration	(43)
circRNA_103809	AP4E1	miR-1270, miR-377-3p	PLAGL2, FGFR1-ERK	28/60 HBV-HCC patients	Promote proliferation, migration, invasion and EMT (37), proliferation,‬ cycle progression, and migration (38)‬	(37–39)
has_circ_0008450	CMTM3	miR-214-3p miR-548p	EZH2 (44)	50/70 HBV-HCC patients (44)	Promote cell viability, migration and invasion, inhibit cell apoptosis	(44, 45)
has_circ-0001955	CSNKIG1	miR-516a-5p mi-R-145-5p	TRAF6, MAPK11 NRAS	34/60 HBV-HCC patients (46)	Promote proliferation, migration, invasion and colony formation	(46, 47)
circUHRF1	UHRF1	miR-449-5p	IFN-γ and TNF-α, TIM-3.	216/240 HBV-HCC patients	Inhibit NK cell functions	(48)
has_circ_0082002	MET	miR-30-5p	CXCL10, DPP4	173/209 HBV-HCC patients	Promote invasion and metastasis, EMT and cancer immunosuppression	(49)
circ_0021093	ST5	miR-766-3p	MTA3	43/82 HBV-HCC patients	Promote cell growth, migration and invasion, inhibit apoptosis.	(50)
		miR-432	ANXA2	50/60 HBV-HCC patients	Promote proliferation, migration, invasion and EMT	(51)
circ_0000267	FAM53B	miR-646	Not investigated	32/59 HBV-HCC patients	Promote cell growth, migration and invasion, inhibit apoptosis	(52)
circ_0005394	ZC3H7A	miR-507 miR-515-5p	E2F3 CXCL6	43/82 HBV-HCC patients	Promote migration and invasion, inhibit apoptosis	(53)
circZEB1.33	ZEB1	miR-200a-3p	CDK6	58/64 HBV-HCC patients	Promote proliferation	(54)
has_circ_0088030	PTGR1	miR449a	MET	63/82 HBV-HCC patients	Promote proliferation, migration and invasion	(8)
has_circ_0066659	TMEM45A	miR-665	IGF2	34/68 HBV-HCC patients	Promote growth phenotype and cell cycle of cancer cells.	(55)
has_circ_0008285	CDYL	miR-892a miR-328-3p	HDGF HIF1AN	10/10microarray analysis. 143/149 HBV-HCC patients	Promote proliferation, self-renewal, chemoresistance, stem-like properties of HCC cells	(56)
has_circ_0070396	NUDT9	Not investigated	Not investigated	108/111 HBV-HCC patients	Not investigated	(57)
circRASGRF2	RASGRF2	miR-1224	Not investigated	35/68 HBV/HCC patients	Promote proliferation, invasion and migration	(58)
circ_0058124	FN1	miR-1205	E2F1	31/64 HBV/HCC patients	Promote proliferation. inhibit apoptosis, suppress the sorafenib sensitivity of HCC cells	(59)
has_circRNA_104348	MAP2K5	miR-187-3p	RTKN2-Wnt-beta-catenin	32/60 HBV/HCC patients	Promote proliferation, migration and invasion, inhibit apoptosis	(60)
circGSK3B	GSK3B	miR-1265	CAB39	31/40 HBV/HCC patients	Promote proliferation, migration, invasion	(61)
circHIPK3	HIPK3	miR-338-3p	ZEB2	All patients are free of HBV and HCV	Promote migration, invasion, metastases and EMT	(62)
has_circ_102559	NUMB	miR-130a-5p	ANXA2	40/74 HBV/HCC patients	Promote proliferation, migration, invasion, metastasis and EMT	(63)
has_circ_104566	PSD3	miR-338-3p	FOXP1	46/87 HBV/HCC patients	Decrease apoptosis and E-cadherin, increase cell viability, proliferation, migration, invasion, and N-cadherin.	(64)
has_circ_0005785	ANAPC7	miR-578	APRIL	41/60 HBV/HCC patients	Promote proliferation and metastasis, inhibit cell cycle arrest and apoptosis	(65)
circRNA_SORE	SORE	Not investigated	YBX1	21/60 HBV/HCC patients	Spread sorafinb resistance among HCC cells by exosome	(66)
		miR-103a-2-5p and miR-660-3p	beta-catenin	Not investigated	Decrease the efficacy of sorafenib-induced resistance	(67)
circIL4R	IL4R	miR-541-3p	GPX4	Not investigated	Promote oncogenesis and inhibit ferroptosis of HCC cells.	(68)
circRNA_cIARS	cIARS	Not investigated	ALKBH5	Not investigated	regulate SF-induced ferroptosis	(69)
circPTK2	PTK2	miR-92a	E-cadherin	Not investigated	enhanced cell proliferation and invasion	(70)
circ-0038718	IL4R	miR-139-3p	Not investigated	Not investigated	Promote proliferation and metastatic ability	(71)
circRNA7	Not recorded	miRNA7-5p	VE-cadherin/Notch4	Not investigated	Promote HCC vasculogenic mimicry	(72)
circUGGT2	UGGT2	miR-526b-5p	RAB1A	Not investigated	Promote proliferation, migration, invasion, colony formation and cell cycle progression	(73)
circRNA_ZFR	ZFR	Not investigated	MAP2K1	Not investigated	Promote proliferation	(74)
has_circ_0091579	GPC3	miR-940	TACR1	Not investigated	Promote cell viability, migration, invasion and colony formation, inhibit cell cycle arrest and apoptosis	(75)
circRNA_ CDR1as	CDR1as	miR-1287	Raf1	Not investigated	Promote proliferation, migration	(76)
circ_BIRC6	BIRC6	miR-877-5p	YWHAZ	Not investigated	The inhibitory of effect of paclitaxil on HCC tumorigenesis	(77)
circ_0091581	GPC3	miR-591	FOSL2	Not investigated	enhance the viability, colony formation, metastasis and cell cycle and inhibit the apoptosis of HCC cells	(78)
circSOD2	SOD2	miR-502-5P	DNMT3A JAK2/STAT3	Not investigated	Promote liver cancer cells growth, migration and cell cycle progression	(79)
circGFRA1	GFRA1	miR-149	Not investigated	Not investigated	Promote proliferation, migration and angiogenic activity	(80)
circ_0008305	PTK2	miR-186	TMED2	Not investigated	Promote proliferation, migration and invasion	(81)
circFAT1	FAT1	miR-30a-5p	REEP3	Not investigated	Promote proliferation and invasion	(82)
has_circ_0006916	HOMER1	miR-599	SRSF2	Not investigated	Promote cell viability, colony formation, migration and invasion. Inhibit cell cycle arrest and apoptosis	(83)
circRNA_001306	MARCH6	miR-584-5p	CDK16	Not investigated	Promote proliferation, inhibit apoptosis	(84)
has_circ_0039053	ITGAL	miR-637	USP21	Not investigated	Promote proliferation and invasion	(85)
circ_0130911	UTRN	Not investigated	CCNB1 OIP5 RACGAP1	Not investigated	Not investigated	(86)
circPUM1	PUM1	miR-1208	MAP3K2	Not investigated	Promote proliferation, migration, invasion and EMT	(87)
circRNA_102272	RTN1	miR-326	RUNX2	Not investigated	Promote proliferation and cisplatin-resistance	(88)
circRNA_ZNF292	ZNF291	Not investigated	Wnt/beta-catenin	Not investigated	Promote proliferation and cell cycle, inhibit apoptosis	(89)
circ_0003998	ARFGEF2	miR-218-5	EIF5A2	Not investigated	Promote resistant cell viability, migration, invasion and EMT, inhibit DOX cytotoxicity	(90)
circRNA_GFRA1	GFRA1	miR-498	NAP1L3	Not investigated	Promote proliferation, migration and invasion	(91)
circ_0001175	YTHDF1	miR-130a-5p	SNX5	Not investigated	Promote proliferation, migration, invasion and lung metastasis	(92)
circWHSC1	WHSC1	miR-142-3p	HOXA1	Not investigated	Promote proliferation, migration, invasion	(93)
has_circ_0061395	BACH1	miR-877-5p	PIK3R3	Not investigated	Promote proliferation, migration, invasion. Inhibit cell cycle, apoptosis	(94)
has-circ-0034326 and has-circ-0011950	OTUD7A HIVEP3	miR-25-3p, miR-3692-5p, and miR-4270	NRAS, ITGA5, SEC14L2, SLC12A5, and SMAD2	Not investigated	Not investigated	(95)
circZFR s	ZFR	miR-375	HMGA2	Not investigated	Promote proliferation, glycolysis, inhibit apoptosis	(96)
circSOX4	SOX4	miR-432	Not investigated	Not investigated	Promote proliferation, migration and invasion, inhibit apoptosis	(97)
circFOXM1	FOXM1	miR-1324	MECP2	Not investigated	Promote proliferation, inhibit apoptosis	(98)
circ_SLIT3	SLIT3	miR-223-3p	CXCR4	Not investigated	Promote proliferation, migration and invasion, inhibit apoptosis	(99)
circ_0031242	PRMT5	miR-924	POU3F2	Not investigated	Promote proliferation, migration and invasion, inhibit apoptosis	(100)
circPVT1	PVT1	miR-377	TRIM23	Not investigated	Promote proliferation and glycolysis, inhibit apoptosis	(101)
has_circ_0004277	WDR37	Not investigated	ZO-1	Not investigated	Promote proliferation, migration and EMT	(102)
circCAMSAP1	CAMSAP1	miR-1294	GRAMD1A	Not investigated	Promote proliferation, migration and invasion	(103)
has_circ_0009910	MFN2	has-miR-455-5p, has-miR-615-3p, has-miR-3926, has-miR-5197-3p, and has-miR-6836-3p	DLGAP5/MCM5 MCM5/MCM6/MCM3/CDC20/CCNB1/CDC7	Not investigated	Not investigated	(104)
has_circ_0049783	CLEC17A	has-miR-18a-3p and has-miR-8071	ZWINT	Not investigated	Not investigated	
has_circ_0089172	NUP214	has-miR-4524a-3p, has-miR-3154, has-miR-3190-5p	CDC7/CCNB1/CENPU/ASPM/ECT2/NDC80	Not investigated	Not investigated	
circ_0008305	PTK2	miR-660	BAG5	Not investigated	Promote proliferation, migration, invasion and cell cycle. Inhibit apoptosis	(105)
circ_LDLR	LDLR	miR-7	RNF38	Not investigated	Promote proliferation, migration, invasion and EMT	(106)
circARNT2	ARNT2	miR-155-5p	PDK1	Not investigated	Promote proliferation, inhibit the cisplatin sensitivity of HCC cells	(107)
has_circ_0067934	PRKCI	miR-1324	FZD5-Wnt-β-catenin	Not investigated	Promotes tumor growth and metastasis	(108)
circMYLK	MYLK	miR-29a (109) miR-362-3p (110)	KMT5C (109) Rab23 (110)	Not investigated	Inhibit proliferation, migration and invasion, promote apoptosis	(109, 110)
has_circ-0000517	RPPH1	miR-326 (111, 112) miR-1296-5p (113)	SMAD6 (111) IGF1R (112) TXDNC5 (113)	Not investigated	Promote proliferation, colony formation, migration, invasion, glycolysis and cell cycle	(111–113)
circPVT1	PVT1	miR-3666 miR-203	SIRT7HOXD3	Not investigated	Promote proliferation, colony formation, migration, inhibit apoptosis	(114, 115)
circ_0015756	CFH	miR-7 (116) miR-610 (117)	FAK (116) FGFR1 (117)	Not investigated	Promote proliferation, migration and invasion, inhibit apoptosis	(17, 116)
has_circ_0084922	KIAA1429	Not investigated	YTHDF3, Zeb1	Not investigated	Promote migration, invasion and EMT	(118)
has_circ_0065964	ABHD14A-ACY1	Not investigated	Not investigated	Not investigated	Not investigated	(119)
has_circ_0011386	EIF3I					
has_circ_0044172	MAP3K14					
has_circ_0010882	RPL1					
Circ_0004194	β-catenin	Not investigated	Wnt/β-catenin	Not investigated	Promote cell growth, migration and colony formation	(120)
has_circRNA_102034	RHOT1	Not investigated	TIP60, NR2F6	Not investigated	Promote proliferation, migration and invasion, inhibit apoptosis	(121)
circ_0016788	TRIM11	Not investigated	Not investigated	Not investigated	Not investigated	(122)
has_circ_0003998	ARFGEF2	Not investigated	Not investigated	Not investigated	Not investigated	(123)
has_circ_104075	VPS13C	miR-582-3p	YAP-HNF4a	Not investigated	Promote tumorigenesis	(124)
has_circ_0008234	FOXP1	miR-875-3p, miR-421	SOX9	Not investigated	Promote proliferation, invasion, inhibit apoptosis	(125)
has_circ_0004001	CLK1	biological pathway analysis	biological pathway analysis	Not investigated	Not investigated	(126)
has_circ_0004123	ETV6					
has_circ_0075792	KDM1B					
has_circ_0000798	BPTF	miRanda v3.3a and RNAhybrid 2.1 predict	miRanda v3.3a and RNAhybrid 2.1 predict	Not investigated	Not investigated	(127)
has_circ_0005505	IRAK3					
has_circ_0001394	TBC1D14					
has_circ_0003258	ZNF652	miR-29a-3p	GUCD1	Not investigated	Promote proliferation, migration, invasion and glycolysis.	(128)
has_circ_0010090	FBLIM1	miR-338	LRP6	Not investigated	Promote tumor growth and glycolysis.	(129)
has_circ_101237	CDK8	Not investigated	Not investigated	Not investigated	Not investigated	(130)
has_circ_0003288	BIRC6	miR-3918	Bcl2	Not investigated	Promote proliferation, migration, invasion, inhibit apoptosis.	(131)
has_circ_0000199	AKT3	Not investigated	Not investigated	Not investigated	Not investigated	(132)
**Down-regulation**						
has_circ_0001649	SHPRH	miR-1283, miR-4310, miR-182-3p, miR-888-3p, miR-4502, miR-6811-5p, miR- 6511b-5p, miR-1972	Not investigated	66/89 HBV-HCC patients	Promotes HCC metastasis	(133)
hsa_circ_0001727	ZKSCAN1	Not investigated	Not investigated	85/102 HBV-HCC patients	Inhibits cell proliferation, migration, and invasion	(134)
circSMYD4	SMYD4	miR-584-5p	Not investigated	27/40 HBV/HCC patients	Inhibit proliferation, migration, invasion. inhibit apoptosis	(135)
circ-0003418	Not traceable	miR-7, miR-383	Wnt/β-catenin	36/46 HBV/HCC patients	Inhibit proliferation, migration, invasion, suppress cisplatin resistance of HCC cells	(136)
hsa_circ_0007456	MAP2K4	miR-6852-3p	ICAM-1	61/72 HBV/HCC patients	inhibit the conjugation in NK cells	(137)
hsa_circ_0004018	SMYD4	miR-30e-5p, miR-647, miR-92a-1-5p, miR-660-3p, miR-626	MYC (miR-30e-5p/miR-626)	86/101 HBV-HCC patients	Inhibit HCC carcinogenesis and metastasis.	(138)
hsa_circ_0003570	FAM53B	Not investigated	Not investigated	90/105 HBV-HCC patients	Inhibit HCC invasion and metastasis	(139)
hsa_circ_0085154	ARSP91	miR-7	ADAR1	69/83 HBV-HCC patients	Inhibits colony formation and tumor growth	(140)
hsa_circ_0001445	SMARCA5	miR-17-3p, miR-181b-5p	TIMP3	166/208 HBV-HCC patients	Inhibits proliferation and migration	(141)
hsa_circ_0001141	ITCH	Not investigated	Not investigated	450/1800 HBV-HCC patients	Not investigated	(142)
hsa_circ_0008717	ABCB10	miR-340-5p/miR-452-5p	NRP1 and ABL2	Not investigated	Inhibit proliferation, colony formation, migration	(143)
circRNA_103809	AP4E1	miR-620	Not investigated	Not investigated	Inhibited HCC cell proliferation, migration and invasion	(37–39)
hsa_circ_0001074	ORC4	miRanda v3.3a and RNAhybrid 2.1 predict	miRanda v3.3a and RNAhybrid 2.1 predict	Not investigated	Not investigated	(127)
hsa_circ_0004771	NRIP1					
hsa_circ_0067735	MED12L					
has_circ_0064428	SLC6A6	Not investigated	Not investigated	Not investigated	Not investigated	(119)
hsa_circ_0055538	RMND5A					
circ_102,166	Not traceable	miR-182, miR-184	FOXO3a, MTSS1, SOX7, p-RB, c-MYC	Not investigated	Inhibit the proliferation, invasion, migration and tumorigenesis of HCC cells	(144)
circ_0004913	TEX2	miR-184	HAMP	Not investigated	Inhibit cell proliferation, migration, and invasion, EMT, and glycolysis in HCC cells	(145)
hsa_circ_0004018	SMYD4	miR-626	DKK3	Not investigated	Inhibit proliferation and migration	(146)
circRNA_101505	RP11-966I7.1	miR-103	NOR1	Not investigated	Suppressed cancer cell growth, enhanced cisplatin toxicity in HCC cells	(147)
circDLC1	DLC1	Not investigated	HuR-MMP1	Not investigated	Inhibit proliferation and motility of hepatoma cells	(148)
circ_0014717	CCT3	miR-668-3p	BTG2	Not investigated	Inhibit proliferation, migration, invasion	(149)
circARPP21	ARPP21	miR-543	LIFR	Not investigated	Inhibit proliferation, migration, invasion	(150)
circPSD3	PSD3	miR-92b-3p	Smad7	Not investigated	Inhibit activation and proliferation of HSCs	(151)
circC3P1	C3P1	miR-4641	PCK1	Not investigated	Inhibit proliferation, migration and invasion	(152)
hsa_circ_0007874	MTO1	miR-9	p21	Not investigated	Inhibits cell proliferation and invasion; promotes apoptosis	(153)
hsa_circ_0005986	PRDM2	miR-129-5p	Notch1	Not investigated	Inhibits cell proliferation and cell cycle progression	(154)
hsa_circ_0051443	TRAPPC6A	miR-331-3p	BAK1	Not investigated	Inhibit proliferation and migration	(155)
circSMARCA5	SMARCA5	Not investigated	Not investigated	Not investigated	Inhibit proliferation, invasion, promote apoptosis	(156)

Undoubtedly, virally-encoded circRNAs (vcircRNAs) have different mechanisms and effects in the regulation of signaling pathways involved in viral infection and oncogenesis between HBV-HCC and non-HBV HCC (157). Nevertheless, the vcircRNA research is in signaling pathways regulation where many puzzles remain to be solved. HBV_circ_1, a recently identified HBV-encoded circRNA, is derived by the intronless pgRNA, which is produced *via* the homologous recombination of the inverted repeat sequences at both 3′ and 5′ ends of the pgRNA, promoting viral replication (12, 158). Particularly, herpesviruses cannot express antigenic viral proteins during the latency in order to escape the host immune surveillance. Due to immunogenicity lack, circRNAs is likely an ideal strategy for the viruses to regulate themselves and the host environment (157). Therefore, we hypothesize various innate and adaptive immune-associated pathways enhance the chronic viral infection and viral replication, and finally tumor initiation. More efforts are warranted to investigate the pathways involved immunoevasion of foreign circRNAs in HBV-HCC.

### Animal Model for circRNAs Study

Various circRNAs are expressed in serum, plasm, liver tissues, liver tumors, liver cancer cells and exosomes. Also, several tumor-bearing mouse models transplanted with circRNAs were used to analyze the detailed mechanisms in HCC development. For example, HCCLM3 cells with or without reduced circUHRF1 were injected into the male NOD/SCID mouse. And then, NK cells were injected intravenously through the tail vein when the tumor reached a volume of approximately 100 mm^3^. The implantation of circUHRF1 knockdown cells resulted in sensitivity to anti-PD1 therapy and overall survival improvement (48). In another experiment, C57BL/6 mice implanted with Hep1–6-circMET cells had a larger tumor burden compared to the controls. Importantly, these experiments showed that the density of tumor-infiltrating CD8^+^ lymphocytes in tumors injected with Hep1–6-control cells was significantly higher (49). A xenograft assays using female BALB/c mice subcutaneously injected HepG2 cells with or without transfection of circ_0008305 siRNA found that downregulation of circ_0008305 repressed HCC tumor growth *in vivo* (105). These circRNAs behaved in tumor-bearing mice could help us further understand the mechanisms in HCC development in depth.

### Several Critical Cell Signaling Pathways Regulated by circRNAs in HBV-HCC

Importantly, the interplay between circRNAs and miRNAs for the regulation of different signaling cascades has enabled us to develop a better understanding of the mechanism of HBV-HCC development (159). For example, A differential expression of the circulating miRNAs from 50 patients diagnosed with chronic HBV infection and hepatic fibrosis based on Scheuer’s staging criteria found the majority of the target genes of the identified miRNAs affected hepatic fibrosis *via* the TGF−/Smad, Wnt, MAPK, Jak/STAT and VEGF pathways (160). As a tumor suppressor, circSMAD2 can remarkably impede TGF/SMAD signaling and epithelial to mesenchymal transition (EMT) by inhibiting microRNA-9 (161). Overexpressed circSMAD2 inhibited migratory and invasive potential of HCC cells and considerably reduced TGFβ1-CircSMAD2 sponging for miR-629 (162). Notably, hsa_circ_0000517 regulated SMAD6 expression through competing endogenous RNA (ceRNA) for miR-326. Up-regulation of AMAD6 overturned the inhibitory impacts of miR-326 mimics on cell proliferation, colony formation, migration, and invasion of HCC cells (112).

Notch signaling pathway facilitates HBV cccDNA transcription *via* triggering PKA-phospho-cAMP response element-binding protein (CREB) cascade and is regulated by E3 ubiquitin ligase-modulation of the Notch intracellular domain (163). Meanwhile, studies have shown that NOTCH pathway is involved in different steps of carcinogenesis of HCC. Hsa_circ_0005986 was associated with chronic hepatitis B infection history. Both hsa_circ_0005986 and Notch1 were targets of miR-129-5p, and that hsa_circ_0005986 knockdown decreased the expression level of Notch1 and accelerated cell proliferation by facilitating the G0/G1 to S phase transition of HepG2 and Huh7 cells (154). In SMMC-7721 cells, high expression of circ-CDYL could promote distinguished rise of survivin and HIF1AN expression levels, and enhance the interactions between NOTCH2 and HIF1AN in SMMC-7721 cells (56).

In recent years, series of studies have provided evidence that the JAK/STAT signaling pathway is closely related to the occurrence and development of liver fibrosis and HCC caused by HBV (164). Some experiments have documented that H3K27ac and H3K4me3 expression modification (active gene transcription hallmarkers), and circSOD2 expression were further increased after histone writer EP300 and WDR5 binding to circSOD2 promoter. On one side, CircSOD2 could promote cell growth, migration, and tumor growth of liver cancer. On the other side, circSOD2 acted as a sponge on miR-502-5p and rescued DNMT3a expression, which could inhibit SOCS3 expression and accelerate JAK2/STAT3, SOCS3 downstream signaling pathway activation. In a feedback way, activated STAT3 regulated circSOD2 expression (79). Circ-LRIG3 worked with EZH2 and STAT3 together and facilitated EZH2-induced STAT3 methylation and activation. In turn, activated STAT3 could positively respond to circ-LRIG3 promoter to facilitate circ-LRIG3 transcription activity. Finally, Circ-LRIG3 promoted malignant biological behavior of HCC cell (165). In additional, circ9119 targeted JAK1/STAT3 in HepG2/Huh-7 cells by competitively binding miR-26a, resulting in less proliferation of HCC cells and increasing apoptosis after circ9119 silence (166).

The Wnt/β-catenin signaling pathway has a key role of the modulation of immune responses and in the orchestration of a chronic low-level inflammation state favoring HCC development infected by HBV (167). Of note, circβ-catenin has higher expression in liver cancer tissues than that in adjacent normal tissues. Also, circβ-catenin could affect a wide spectrum of Wnt pathway-related genes. 370-amino acid β-catenin isoform can activate the Wnt pathway by antagonizing GSK3β-induced β-catenin phosphorylation and degradation. In the nude mice injected with circβ-catenin-silenced Huh7 cells, tumors were smaller in size and had a marked reduction in the pulmonary metastatic lesions (120). Similarly, tumor growth was remarkably reduced in mice transplanted with circFBLIM1-silenced Huh7 cancer cells. Here, circFBLIM1 acted as a sponge for miR-338 and promoted HCC progression *via* targeting LRP6 (129). In sorafenib-resistant HCC cells, circRNA-SORE sequestered miR-660–3p and miR-103a-2-5p-mediated targeting of Wnt2b and β-catenin pathway and inducing sorafenib resistance. This was involved in an increased level of N6-methyladenosine (m6A) at a specific adenosine in circRNA-SORE (67).

## Therapeutic Strategies for HBV-HCC Involving circRNAs

Given the association of different circRNA expression patterns with HBV-HCC, emerging evidence indicates that both tissue and circulating circRNAs may serve as potential biomarkers for diagnostic, prognostic and therapeutic purposes (**Table 1**).

### circRNA Diagnostic Biomarkers in HBV-HCC

Currently, effective biomarkers for early and accurate diagnosis of HBV-HCC are still lacking. As classic diagnostic biomarkers, α-fetoprotein (AFP), AFP-L3, and desgamma-carboxyprothrombin (DCP) are only modestly beneficial in diagnosis of HCC. Due to their higher stability and abundance in HCC, circRNAs may be perfect diagnostic indicators, especially in AFP-normal HCC patients. Combined with AFP, three circulating circRNAs (circ_0009582, circ_0037120 and circ_0140117) were reported to have higher sensitivity and specificity as potential diagnostic biomarkers for predicting HBV-HCC occurrence (18). The risk score analysis with the ROC curve in the training set and validation set showed values of 0.988 and 0.955, respectively. According to a circRNA microarray analysis, Zhu et al. (20) found that plasma hsa_circ_0027089 exhibited the highest significance and further distinguished HBV-HCC patients from non-HCC patients. The combination of hsa_circ_0027089 and AFP had better sensitivity but poorer specificity in HBV-HCC than in cirrhotic, healthy and non-HCC patients. Yu et al. built an HBV-HCC diagnostic model, CircPanel, containing three circRNAs (hsa_circ_0000976, hsa_circ_0007750 and hsa_circ_0139897). In addition, CircPanel+AFP was calculated as Logit = −2.152 + 3.321 × CircPanel+2.241 × AFP. They found that both CircPanel and CircPanel+AFP showed a higher accuracy than AFP alone in distinguishing individuals with HBV-HCC from those with non-HCC liver disease. Furthermore, both CircPanel and CircPanel+AFP performed well in detecting small HCC lesions (≤3 cm) and AFP-negative HBV-HCC, indicating the high diagnostic value of hsa_circ_0000798 in HBV-HCC (19).

### circRNA Prognostic Biomarkers in HBV-HCC

To date, dozens of noncoding RNAs (ncRNAs) have been reported to have essential roles in HCC progression and to be potential prognostic biomarkers of HBV-HCC. For example, a miRNA panel including seven miRNAs provided high diagnostic accuracy for HBV-HCC (168). circRNAs are newly classified endogenous ncRNA members that have been identified as outcome predictors for patients with HBV-HCC in some studies. Huang et al. (169) revealed that an elevated circRNA-100338/miR-141-3p/RHEB axis was involved in activation of the mTOR signaling pathway in HCC. Clinical specimen analysis indicated that circRNA-100338 was upregulated in HCC tissues, which also showed an increased RHEB RNA level. Correlation analysis of RHEB expression with the clinicopathological parameters of HBV-HCC patients suggested that circRNA-100338 was an indicator of poor prognosis in HBV-HCC. Clinically, high expression of circ-ARL3 was observed in HBV+ HCC tissues compared to HBV−HCC tissues. circ-ARL3 expression was positively associated with HBsAg+ status, and in HBV-HCC patients, a high circ-ARL3 expression level was related to a shorter survival time than observed in patients with a low circ-ARL3 expression level (16), verifying its ability to predict the prognosis of patients with HBV-HCC.

### circRNA Therapeutic Biomarkers in HBV-HCC

Recently, some circRNAs have been demonstrated to regulate gene expression *via* circRNA-miRNA-mRNA interaction networks to facilitate HBV-HCC hepatocarcinogenesis and thus might be useful in guiding HCC treatment decisions. In addition to being diagnostic and prognostic biomarkers, these circRNAs can also be used as targets for HCC clinical intervention. As mentioned above, the crucial antagonistic roles of circRNA_100338 and miR-141-3p in the regulation of metastatic potential in HBV-HCC have been confirmed (15). Based on computational analyses followed by experimental verification, circRNA_100338 can directly interact with miR-141-3p in the context of HCC, thus mediating downstream gene regulation in HCC. This indicates that circRNA_100338 could potentially be used as a target in HBV-HCC clinical treatments. Rao et al. (16) found that knockdown of circ-ARL3 suppressed HBV-positive cell proliferation and invasion, whereas these effects were inhibited by silencing of miR-1305, suggesting that the circ-ARL3/miR-1305 regulatory axis exists in HCC cells and may be a promising treatment target for patients with HBV-HCC. Additionally, Jiang et al. (17) found that circ-ATP5H was remarkably expressed in HBV-HCC tissues compared to adjacent noncancer tissues (P<0.0001). Moreover, the expression level of circ-ATP5H was significantly increased in HBV-specific cells compared to HCC cells. These results suggest that circ-ATP5H could be a new biomarker for HBV-HCC treatment.

### Roles of circRNA in HCC Drug Resistance

At present, chemotherapy and immunotherapy agents for advanced HCC are greatly limited by drug resistance, leading to cancer relapse and intractable tumors. Mechanistically, the efflux of hydrophobic cytotoxic drugs by cancer cells and induced cell apoptosis contribute to this resistance (170). Recently, the role of circRNAs in HCC drug resistance has become a focus of research in this field (**Table 3**). For instance, circ_0003418 not only exerts an antitumorigenic role in HCC but also facilitates the sensitivity of HCC cells to cisplatin by restraining the Wnt/β-catenin pathway (136). Similarly, the circRNA_101505 expression level is decreased in cisplatin-resistant HCC tissues and cell lines, and circRNA_101505 can sensitize HCC cells to cisplatin by promoting the miR-103/oxidored-nitro domain-containing protein 1 (NOR1) pathway (147). In contrast, circARNT2 is significantly upregulated in HCC tissues and cell lines and facilitates HCC progression *in vivo*. This circRNA suppresses the sensitivity of HCC cells to cisplatin through the miR-155-5p/PDK1 pathway (107). circRNA_102272 may facilitate HCC cisplatin resistance by regulating the miR-326-RUNX2 axis (88). Similarly, silencing of circ_0031242 can mitigate cisplatin resistance while enhancing cisplatin sensitivity. circ_0031242 can also suppress cell viability, migration, and invasion and promote the apoptosis of cisplatin-resistant HCC cells by directly interacting with miR-924 and modulating POU3F2 expression (100). Resistance to doxorubicin, another chemotherapy agent, can be enhanced in HCC cells by the circ_0003998/miR-218-5p/EIF5A2 axis (90). Notably, acquisition of sorafenib resistance is a primary limitation of sorafenib-based chemotherapy. circRNA-SORE hampers YBX1 nuclear interaction with the E3 ubiquitin ligase PRP19 and thus blocks PRP19-mediated YBX1 degradation, which mediates sorafenib resistance in HCC cells (66). circFN1 was demonstrated to mediate sorafenib resistance in HCC cells by sponging miR-1205 and promoting E2F1 expression (90). A mechanistic study of circRNA-SORE found that it sequestered miR-103a-2-5p and miR-660-3p by acting as a microRNA sponge, thereby activating the Wnt/β-catenin pathway and inducing sorafenib resistance (83). Additionally, some patients who receive immune checkpoint therapy do not show a durable or gratifying response. In 2020, two studies showed that dysregulation of certain circRNAs in HCC contributes to immunosuppression. Zhang et al. reported that tumor-derived exosomal circUHRF1 induced natural killer cell exhaustion by upregulating the expression of TIM-3 *via* degradation of miR-449c-5p, thereby driving resistance to anti-PD1 immunotherapy in HCC patients (48). Another study found that circMET promoted HCC progression by inducing epithelial to mesenchymal transition and enhancing immunosuppression and anti-PD1 therapy resistance through regulation of the miR-30-5p/Snail/dipeptidyl peptidase 4 (DPP4)/CXCL10 axis (49). Although there are few studies on the molecular function of circRNAs in chemotherapy and immunotherapy agents, especially for HBV-HCC, the mechanisms and roles of circRNAs in drug resistance must be mined to advance HBV-HCC treatment, which may offer better approaches to reverse chemoresistance and immune resistance.

**Table 3:** Deregulation and roles of circular RNAs in drug resistance of hepatocellular carcinoma.

CircRNA	Gene symbol	miR Target	miR target genes/proteins	Deregulation	Drug	Ref
**Inhibiting drug resistance**						
circ-0003418	Not traceable	miR-7, miR-383	Wnt/β-catenin	Down	cisplatin	(136)
circRNA_101505	RP11-966I7.1	miR-103	NOR1	Down	cisplatin	(147)
**Promoting drug resistance**						
circARNT2	ARNT2	miR-155-5p	PDK1	Up	cisplatin	(107)
circ_0031242	PRMT5	miR-924	POU3F2	Up	cisplatin	(100)
circRNA_102272	RTN1	miR-326	RUNX2	Up	cisplatin	(88)
circ_0003998	ARFGEF2	miR-218-5	EIF5A2	Up	doxorubicin	(90)
circ_0058124	FN1	miR-1205	E2F1	Up	sorafenib	(59)
circRNA-SORE	SORE	Not investigated	YBX1	Up	sorafenib	(66)
circUHRF1	SORE	miR-103a-2-5p and miR-660-3p	β-catenin	Up	sorafenib	(67)
	UHRF1	miR-449c-5p	TIM-3	Up	PD-1	(48)
circMET	MET	miR-30-5p	Snail/DPP4/CXCL10	Up	PD-1	(49)

## Conclusion and Perspectives

With the rapid development of advanced experimental techniques, including next-generation sequencing technology and bioinformatics tools, allowing the characterization of novel molecular biology circRNAs associated with HBV-HCC, circRNAs are being increasingly identified and attracting increasing attention from researchers worldwide. CHB-related circRNA-miRNA-mRNA pathway analyses have revealed that dysregulated circRNAs are correlated with CHB and regulate HBV replication. As multifaceted regulators, circRNAs contribute to regulation of gene expression and signaling pathways and to translation of proteins directly *via* the miRNA-mRNA axis. Therefore, circRNAs induce aberrant functions in the tumor microenvironment and can become novel biomarkers for HBV-HCC diagnosis, prognosis determination and treatment response. Recently, an increasing number of circRNAs have been found to participate in HCC drug resistance, and the involved molecular biology mechanisms are gradually being revealed. However, knowledge of the emerging functions of circRNAs in drug resistance or other aspects of HCC development is only the tip of the iceberg, and their roles in HBV-HCC are still unclear. In the future, targeting dysregulated endogenous circRNAs may be a promising way to reverse drug resistance. circRNAs from a potential RNA virus may act as new tumor antigens for HBV-HCC vaccines and oncolytic viruses to activate or induce antitumor immunity. Further in-depth translational research and clinical trials are urgently needed and may ultimately open potential approaches for antitumor therapy for HBV-HCC.

## Author Contributions

RL and PH performed the majority of the writing, prepared the figures and tables. LL and JZ performed data accusation and writing. XW performed data accusation. All authors contributed to the article and approved the submitted version.

## Funding

This study was supported by Science and Technology Research Program of Chongqing Municipal Education Commission (No. KJQN201800416), Basic and Advanced Research Project of Science and Technology Commission of Chongqing Municipality (No. cstc2018jcyjAX0162), and Science and Health Joint Research Project of Chongqing Municipality (2020GDRC013).

## Conflict of Interest

The authors declare that the research was conducted in the absence of any commercial or financial relationships that could be construed as a potential conflict of interest.

## References

[1] Villanueva A. Hepatocellular Carcinom. N Engl J Med (2019) 380(15):1450–62. 10.1056/NEJMra1713263 30970190

[2] Liao R Zhang XD Li GZ Qin KL Yan X. Comparison of Transcatheter Arterial Chemoembolization With Raltitrexed Plus Liposomal Doxorubicin *vs.* Tegafur Plus Pirarubicin for Unresectable Hepatocellular Carcinoma. J Gastrointest Oncol (2020) 11(4):747–59. 10.21037/jgo-20-59 PMC7475337 32953158

[3] He M Li Q Zou R Shen J Fang W Tan G. Sorafenib Plus Hepatic Arterial Infusion of Oxaliplatin, Fluorouracil, and Leucovorin *vs.* Sorafenib Alone for Hepatocellular Carcinoma With Portal Vein Invasion: A Randomized Clinical Tria. JAMA Oncol (2019) 5(7):953–60. 10.1001/jamaoncol.2019.0250 PMC6512278 31070690

[4] Bach DH Lee SK Sood AK. Circular RNAs in Cance. Mol Ther Nucleic Acids (2019) 16:118–29. 10.1016/j.omtn.2019.02.005 PMC6411617 30861414

[5] Qiu L Xu H Ji M Shang D Lu Z Wu Y. Circular RNAs in Hepatocellular Carcinoma: Biomarkers, Functions and Mechanisms. Life Sci (2019) 231:116660. 10.1016/j.lfs.2019.116660 31319086

[6] Di Timoteo G Dattilo D Centron-Broco A Colantoni A Guarnacci M Rossi F. Modulation of circRNA Metabolism by M(6)A Modificatio. Cell Rep (2020) 31(6):107641. 10.1016/j.celrep.2020.107641 32402287

[7] Patop IL Wust S Kadener S. Past, Present, and Future of circRNAs. EMBO J (2019) 38(16):e100836. 10.15252/embj.2018100836 31343080 PMC6694216

[8] Wang G Liu W Zou Y Deng Y Luo J Zhang Y. Three Isoforms of Exosomal Circptgr1 Promote Hepatocellular Carcinoma Metastasis *via* the Mir449a-MET Pathway. EBioMedicine (2019) 40:432–45. 10.1016/j.ebiom.2018.12.062 PMC6412851 30630697

[9] Nassal M. HBV cccDNA: Viral Persistence Reservoir and Key Obstacle for a Cure of Chronic Hepatitis B. Gut (2015) 64(12):1972–84. 10.1136/gutjnl-2015-309809 26048673

[10] Tan KE Lim YY. Viruses Join the Circular RNA World. FEBS J (2020). 10.1111/febs.15639 PMC7753765 33236482

[11] Zhou TC Li X Chen LJ Fan JH Lai X Tang Y. Differential Expression Profile of Hepatic Circular RNAs in Chronic Hepatitis B. J Viral Hepat (2018) 25(11):1341–51. 10.1111/jvh.12944 29888838

[12] Sekiba K Liang Z Otsuka M Kishikawa T Yamagami M Suzuki T. DHX9 Regulates Production of Hepatitis B Virus-Derived Circular RNA and Viral Protein Levels. Oncotarget (2018) (30):20953–64. 10.18632/oncotarget.25104 PMC5940377 29765512

[13] Zhang L Wang Z. Circular RNA Hsa_Circ_0004812 Impairs IFN-Induced Immune Response by Sponging miR-1287-5p to Regulate FSTL1 in Chronic Hepatitis B. Virol J (2020) 17(1):40. 10.1186/s12985-020-01314-0 32188476 PMC7079541

[14] Wang M Yu F Li P. Circular RNAs: Characteristics, Function and Clinical Significance in Hepatocellular Carcinom. Cancers (Basel) (2018) 10(8):258. 10.3390/cancers10080258 PMC6116001 30072625

[15] Huang XY Huang ZL Xu YH Zheng Q Chen Z Song W. Comprehensive Circular RNA Profiling Reveals the Regulatory Role of the circRNA-100338/miR-141-3p Pathway in Hepatitis B-Related Hepatocellular Carcinoma. Sci Rep (2017) 7(1):5428. 10.1038/s41598-017-05432-8 28710406 PMC5511135

[16] Rao X Lai L Li X Wang L Li A Yang Q. N(6) -Methyladenosine Modification of Circular RNA Circ-ARL3 Facilitates Hepatitis B Virus-Associated Hepatocellular Carcinoma *via* Sponging miR-1305. IUBMB Life (2021) 73(2):408–17. 10.1002/iub.2438 33372396

[17] Jiang W Wang L Zhang Y Li H. Circ-ATP5H Induces Hepatitis B Virus Replication and Expression by Regulating miR-138-5p/TNFAIP3 Axi. Cancer Manag Res (2020) 12:11031–40. 10.2147/CMAR.S272983 PMC7648158 33173336

[18] Wu C Deng L Zhuo H Chen X Tan Z Han S. Circulating circRNA Predicting the Occurrence of Hepatocellular Carcinoma in Patients With HBV Infection. J Cell Mol Med (2020) 24(17):10216–22. 10.1111/jcmm.15635 PMC7520265 32692470

[19] Yu J Ding WB Wang MC Guo XG Xu J Xu QG. Plasma Circular RNA Panel to Diagnose Hepatitis B Virus-Related Hepatocellular Carcinoma: A Large-Scale, Multicenter Study. Int J Cancer (2020) 146(6):1754–63. 10.1002/ijc.32647 31456215

[20] Zhu K Zhan H Peng Y Yang L Gao Q Jia H. Plasma Hsa_Circ_0027089 Is a Diagnostic Biomarker for Hepatitis B Virus-Related Hepatocellular Carcinoma. Carcinogenesis (2020) 41(3):296–302. 10.1093/carcin/bgz154 31535687 PMC7221502

[21] Wang S Cui S Zhao W Qian Z Liu H Chen Y. Screening and Bioinformatics Analysis of Circular RNA Expression Profiles in Hepatitis B-Related Hepatocellular Carcinoma. Cancer Biomark (2018) 22(4):631–40. 10.3233/CBM-170910 PMC13078493 29914004

[22] Arzumanyan A Friedman T Ng IO Clayton MM Lian Z Feitelson MA. Does the Hepatitis B Antigen HBx Promote the Appearance of Liver Cancer Stem Cells? Cancer Res (2011) 71(10):3701–8. 10.1158/0008-5472.CAN-10-3951 PMC3096741 21464043

[23] Zhu M Li W Lu Y Dong X Lin B Chen Y. HBx Drives Alpha Fetoprotein Expression to Promote Initiation of Liver Cancer Stem Cells Through Activating PI3K/AKT Signal Pathway. Int J Cancer (2017) 140(6):1346–55. 10.1002/ijc.30553 27925189

[24] Zhao Y Xu Z Zhou J Yang H. Mir141 Inhibits Proliferation, Migration and Invasion in Human Hepatocellular Carcinoma Cells by Directly Downregulating Tgfbetar1. Oncol Rep (2019) 42(5):1656–66. 10.3892/or.2019.7325 31545479

[25] Lou G Dong X Xia C Ye B Yan Q Wu S. Direct Targeting Sperm-Associated Antigen 9 by miR-141 Influences Hepatocellular Carcinoma Cell Growth and Metastasis *via* JNK Pathway. J Exp Clin Cancer Res (2016) 35:14. 10.1186/s13046-016-0289-z 26790956 PMC4721207

[26] Lin L Liang H Wang Y Yin X Hu Y Huang J. microRNA-141 Inhibits Cell Proliferation and Invasion and Promotes Apoptosis by Targeting Hepatocyte Nuclear Factor-3beta in Hepatocellular Carcinoma Cells. BMC Cancer (2014) 14:879. 10.1186/1471-2407-14-879 25425543 PMC4289273

[27] Liu Y Ding Y Huang J Wang S Ni W Guan J. MiR-141 Suppresses the Migration and Invasion of HCC Cells by Targeting Tiam1. PloS One (2014) 9(2):e88393. 10.1371/journal.pone.0088393 24551096 PMC3923786

[28] Huang XY Huang ZL Xu B Chen Z Re TJ Zheng Q. Elevated MTSS1 Expression Associated With Metastasis and Poor Prognosis of Residual Hepatitis B-Related Hepatocellular Carcinoma. J Exp Clin Cancer Res (2016) 35(1):85. 10.1186/s13046-016-0361-8 27230279 PMC4881066

[29] Han Q Wang X Liao X Han C Yu T Yang C. Diagnostic and Prognostic Value of WNT Family Gene Expression in Hepatitis B Virusrelated Hepatocellular Carcinoma. Oncol Rep (2019) 42(3):895–910. 10.3892/or.2019.7224 31322232 PMC6667889

[30] Wei X You X Zhang J Zhou C. MicroRNA-1305 Inhibits the Stemness of LCSCs and Tumorigenesis by Repressing the UBE2T-Dependent Akt-Signaling Pathwa. Mol Ther Nucleic Acids (2019) 16:721–32. 10.1016/j.omtn.2019.04.013 PMC6535505 31128423

[31] Cao H Chen X Wang Z Wang L Xia Q Zhang W. The Role of MDM2-P53 Axis Dysfunction in the Hepatocellular Carcinoma Transformation. Cell Death Discov (2020) 6(1):53. 10.1038/s41420-020-0287-y 32595984 PMC7305227

[32] Li B Chen Y Wang F Guo J Fu W Li M. Bmi1 Drives Hepatocarcinogenesis by Repressing the TGFbeta2/SMAD Signalling Axis. Oncogene (2020) 39(5):1063–79. 10.1038/s41388-019-1043-8 31591477

[33] Khattar E Kumar P Liu CY Akincilar SC Raju A Lakshmanan M. Telomerase Reverse Transcriptase Promotes Cancer Cell Proliferation by Augmenting tRNA Expression. J Clin Invest (2016) 126(10):4045–60. 10.1172/JCI86042 PMC5096818 27643433

[34] Huang G Li L Liang C Yu F Teng C Pang Y. Upregulated UCA1 Contributes to Oxaliplatin Resistance of Hepatocellular Carcinoma Through Inhibition of miR-138-5p and Activation of AKT/mTOR Signaling Pathway. Pharmacol Res Perspect (2021) 9(1):e00720. 10.1002/prp2.720 33565716 PMC7874507

[35] Yang G Guo S Liu HT. MiR-138-5p Predicts Negative Prognosis and Exhibits Suppressive Activities in Hepatocellular Carcinoma HCC by Targeting FOXC1. Eur Rev Med Pharmacol Sci (2020) 24(17):8788–800. 10.26355/eurrev_202009_22817 32964967

[36] Wang CM Wang Y Fan CG Xu FF Sun WS Liu YG. miR-29c Targets TNFAIP3, Inhibits Cell Proliferation and Induces Apoptosis in Hepatitis B Virus-Related Hepatocellular Carcinoma. Biochem Biophys Res Commun (2011) 411(3):586–92. 10.1016/j.bbrc.2011.06.191 21763284

[37] Cao Y Tao Q Kao X Zhu X. Hsa-circRNA-103809 Promotes Hepatocellular Carcinoma Development *via* MicroRNA-1270/PLAG1 Like Zinc Finger 2 Axi. Dig Dis Sci (2020) 66(5):1524–32. 10.1007/s10620-020-06416-x 32683589

[38] Zhan W Liao X Chen Z Li L Tian T Yu L. Circular RNA Hsa_circRNA_103809 Promoted Hepatocellular Carcinoma Development by Regulating miR-377-3p/FGFR1/ERK Axis. J Cell Physiol (2020) 235(2):1733–45. 10.1002/jcp.29092 31317555

[39] Li X Shen M. Circular RNA Hsa_Circ_103809 Suppresses Hepatocellular Carcinoma Proliferation and Invasion by Sponging miR-620. Eur Rev Med Pharmacol Sci (2019) 23(2):555–66. 10.26355/eurrev_201902_16868 30720163

[40] Xu L Zhang M Zheng X Yi P Lan C Xu M. The Circular RNA ciRS-7 (Cdr1as) Acts as a Risk Factor of Hepatic Microvascular Invasion in Hepatocellular Carcinoma. J Cancer Res Clin Oncol (2017) 143(1):17–27. 10.1007/s00432-016-2256-7 27614453 PMC11819007

[41] Yu L Gong X Sun L Zhou Q Lu B Zhu L. The Circular RNA Cdr1as Act as an Oncogene in Hepatocellular Carcinoma Through Targeting miR-7 Expressio. PloS One (2016) 11(7):e0158347. 10.1371/journal.pone.0158347 27391479 PMC4938625

[42] Shang X Li G Liu H Li T Liu J Zhao Q. Comprehensive Circular RNA Profiling Reveals That Hsa_Circ_0005075, a New Circular RNA Biomarker, Is Involved in Hepatocellular Crcinoma Developmen. Med (Baltimore) (2016) 95(22):e3811. 10.1097/MD.0000000000003811 PMC4900729 27258521

[43] Chen G Shi Y Liu M Sun J. Circhipk3 Regulates Cell Proliferation and Migration by Sponging miR-124 and Regulating AQP3 Expression in Hepatocellular Carcinoma. Cell Death Dis (2018) 9(2):175. 10.1038/s41419-017-0204-3 29415990 PMC5833724

[44] Zhang J Chang Y Xu L Qin L. Elevated Expression of Circular RNA Circ_0008450 Predicts Dismal Prognosis in Hepatocellular Carcinoma and Regulates Cell Proliferation, Apoptosis, and Invasion *via* Sponging miR-548p. J Cell Biochem (2019) 120(6):9487–94. 10.1002/jcb.28224 30556306

[45] Lin T Dai Y Guo X Chen W Zhao J Cao L. Silencing Of Hsa_Circ_0008450 Represses Hepatocellular Carcinoma Progression Through Regulation Of microRNA-214-3p/EZH2 Axi. Cancer Manag Res (2019) 11:9133–43. 10.2147/CMAR.S222716 PMC6817349 31695501

[46] Ding B Fan W Lou W. Hsa_Circ_0001955 Enhances In Vitro Proliferation, Migration, and Invasion of HCC Cells Through miR-145-5p/NRAS Axi. Mol Ther Nucleic Acids (2020) 22:445–55. 10.1016/j.omtn.2020.09.007 PMC7554323 33230448

[47] Yao Z Xu R Yuan L Xu M Zhuang H Li Y. Circ_0001955 Facilitates Hepatocellular Carcinoma (HCC) Tumorigenesis by Sponging miR-516a-5p to Release TRAF6 and MAPK11. Cell Death Dis (2019) 10(12):945. 10.1038/s41419-019-2176-y 31822654 PMC6904727

[48] Zhang PF Gao C Huang XY Lu JC Guo XJ Shi GM. Cancer Cell-Derived Exosomal Circuhrf1 Induces Natural Killer Cell Exhaustion and may Cause Resistance to Anti-PD1 Therapy in Hepatocellular Carcinoma. Mol Cancer (2020) 19(1):110. 10.1186/s12943-020-01222-5 32593303 PMC7320583

[49] Huang XY Zhang PF Wei CY Peng R Lu JC Gao C. Circular RNA circMET Drives Immunosuppression and Anti-PD1 Therapy Resistance in Hepatocellular Carcinoma *via* the miR-30-5p/Snail/DPP4 Axis. Mol Cancer (2020) 19(1):92. 10.1186/s12943-020-01213-6 32430013 PMC7236145

[50] Liu L Qi X Gui Y Huo H Yang X Yang L. Overexpression of Circ_0021093 Circular RNA Forecasts an Unfavorable Prognosis and Facilitates Cell Progression by Targeting the miR-766-3p/MTA3 Pathway in Hepatocellular Carcinoma. Gene (2019) 714:143992. 10.1016/j.gene.2019.143992 31330234

[51] Wang Y Xu W Zu M Xu H. Circular RNA Circ_0021093 Regulates miR-432/Annexin A2 Pathway to Promote Hepatocellular Carcinoma Progression. Anticancer Drugs (2021) 32(5):484–95. 10.1097/CAD.0000000000001053 33675609

[52] Pan H Tang L Jiang H Li X Wang R Gao J. Enhanced Expression of Circ_0000267 in Hepatocellular Carcinoma Indicates Poor Prognosis and Facilitates Cell Progression by Sponging miR-646. J Cell Biochem (2019). 10.1002/jcb.28411 30719761

[53] Sun C Li G Liu MA Novel Circular RNA. Circ_0005394, Predicts Unfavorable Prognosis and Contributes to Hepatocellular Carcinoma Progression by Regulating miR-507/E2F3 and miR-515-5p/CXCL6 Signaling Pathway. Onco Targets Ther (2020) 13:6171–80. 10.2147/OTT.S256238 PMC7334013 32636641

[54] Gong Y Mao J Wu D Wang X Li L Zhu L. Circ-ZEB1.33 Promotes the Proliferation of Human HCC by Sponging miR-200a-3p and Upregulating CDK6. Cancer Cell Int (2018) 18:116. 10.1186/s12935-018-0602-3 30123094 PMC6090603

[55] Zhang T Jing B Bai Y Zhang Y Yu H. Circular RNA Circtmem45a Acts as the Sponge of MicroRNA-665 to Promote Hepatocellular Carcinoma Progressio. Mol Ther Nucleic Acids (2020) 22:285–97. 10.1016/j.omtn.2020.08.011 PMC7516192 33230434

[56] Wei Y Chen X Liang C Ling Y Yang X Ye X. A Noncoding Regulatory RNAs Network Driven by Circ-CDYL Acts Specifically in the Early Stages Hepatocellular Carcinom. Hepatology (2020) 71(1):130–47. 10.1002/hep.30795 31148183

[57] Lyu L Yang W Yao J Wang H Zhu J Jin A. The Diagnostic Value of Plasma Exosomal Hsa_Circ_0070396 for Hepatocellular Carcinoma. Biomark Med (2021) 15(5):359–71. 10.2217/bmm-2020-0476 33666515

[58] Wu D Xia A Fan T Li G. Circrasgrf2 Functions as an Oncogenic Gene in Hepatocellular Carcinoma by Acting as a miR-1224 Sponge. Mol Ther Nucleic Acids (2021) 23:13–26. 10.1016/j.omtn.2020.10.035 33312757 PMC7711184

[59] Yang C Dong Z Hong H Dai B Song F Geng L. Circfn1 Mediates Sorafenib Resistance of Hepatocellular Carcinoma Cells by Sponging miR-1205 and Regulating E2F1 Expressio. Mol Ther Nucleic Acids (2020) 22:421–33. 10.1016/j.omtn.2020.08.039 PMC7533358 33230446

[60] Huang G Liang M Liu H Huang J Li P Wang C. CircRNA Hsa_circRNA_104348 Promotes Hepatocellular Carcinoma Progression Through Modulating miR-187-3p/RTKN2 Axis and Activating Wnt/beta-Catenin Pathway. Cell Death Dis (2020) 11(12):1065. 10.1038/s41419-020-03276-1 33311442 PMC7734058

[61] Li K Cao J Zhang Z Chen K Ma T Yang W. Circular RNA Circgsk3b Promotes Cell Proliferation, Migration, and Invasion by Sponging miR-1265 and Regulating CAB39 Expression in Hepatocellular Carcinom. Front Oncol (2020) 10:598256. 10.3389/fonc.2020.598256 33262952 PMC7688052

[62] Li W Xue H Li Y Li P Ma F Liu M. HIPK3 Circular RNA Promotes Metastases of HCC Through Sponging miR-338-3p to Induce ZEB2 Expression. Dig Dis Sci (2020). 10.1007/s10620-020-06688-3 33247421

[63] Li J Yu Z Zhu Q Tao C Xu Q. Hsa_Circ_102559 Acts as the Sponge of miR-130a-5p to Promote Hepatocellular Carcinoma Progression Through Regulation of ANXA2. Cell Transplant (2020) 29:963689720968748. 10.1177/0963689720968748 33121269 PMC7784593

[64] Liu G Guo W Rao M Qin J Hu F Li K. circRNA Hsa_Circ_104566 Sponged miR-338-3p to Promote Hepatocellular Carcinoma Progressio. Cell Transplant (2020) 29:963689720963948. 10.1177/0963689720963948 33028110 PMC7784580

[65] Wu A Li Y Kong M Zhu B Liu R Bao F. Upregulated Hsa_Circ_0005785 Facilitates Cell Growth and Metastasis of Hepatocellular Carcinoma Through the miR-578/APRIL Axi. Front Oncol (2020) 10:1388. 10.3389/fonc.2020.01388 32974140 PMC7466587

[66] Xu J Ji L Liang Y Wan Z Zheng W Song X. CircRNA-SORE Mediates Sorafenib Resistance in Hepatocellular Carcinoma by Stabilizing YBX1. Signal Transduct Target Ther (2020) 5(1):298. 10.1038/s41392-020-00375-5 33361760 PMC7762756

[67] Xu J Wan Z Tang M Lin Z Jiang S Ji L. N(6)-Methyladenosine-Modified CircRNA-SORE Sustains Sorafenib Resistance in Hepatocellular Carcinoma by Regulating Beta-Catenin Signaling. Mol Cancer (2020) 19(1):163. 10.1186/s12943-020-01281-8 33222692 PMC7681956

[68] Xu Q Zhou L Yang G Meng F Wan Y Wang L. CircIL4R Facilitates the Tumorigenesis and Inhibits Ferroptosis in Hepatocellular Carcinoma by Regulating the miR-541-3p/GPX4 Axis. Cell Biol Int (2020) 44(11):2344–56. 10.1002/cbin.11444 32808701

[69] Liu Z Wang Q Wang X Xu Z Wei X Li J. Circular RNA cIARS Regulates Ferroptosis in HCC Cells Through Interacting With RNA Binding Protein ALKBH5. Cell Death Discov (2020) 6:72. 10.1038/s41420-020-00306-x 32802409 PMC7414223

[70] Gong TT Sun FZ YC J Liu JF Yan Y Li D. The Circular RNA Circptk2 Inhibits EMT in Hepatocellular Carcinoma by Acting as a ceRNA and Sponging miR-92a to Upregulate E-Cadherin. Eur Rev Med Pharmacol Sci (2020) 24(18):9333–42. 10.26355/eurrev_202009_23015 33015774

[71] Sun R Li H Li J Shen S Cui G Dong G. CircRNA Circ-0038718 Promotes Hepatocellular Carcinoma Progression Through Sponging miR-139-3p. Biochem Biophys Res Commun (2020) 533(4):845–52. 10.1016/j.bbrc.2020.07.035 33008587

[72] Bao S Jin S Wang C Tu P Hu K Lu J. Androgen Receptor Suppresses Vasculogenic Mimicry in Hepatocellular Carcinoma *via* Circrna7/Mirna7-5p/VE-Cadherin/Notch4 Signalling. J Cell Mol Med (2020) 24(23):14110–20. 10.1111/jcmm.16022 PMC7754040 33118329

[73] Kong Q Fan Q Ma X Li J Ma R. CircRNA Circuggt2 Contributes to Hepatocellular Carcinoma Development *via* Regulation of the miR-526b-5p/RAB1A Axi. Cancer Manag Res (2020) 12:10229–41. 10.2147/CMAR.S263985 PMC7571581 33116877

[74] Cedric BC Souraka TDM Feng YL Kisembo P Tu JC. CircRNA ZFR Stimulates the Proliferation of Hepatocellular Carcinoma Through Upregulating MAP2K1. Eur Rev Med Pharmacol Sci (2020) 24(19):9924–31. 10.26355/eurrev_202010_23203 33090396

[75] Jiang P Han W Fu Y Chen Q. The Hsa_circ_0091579/miR-940/TACR1 Axis Regulates the Development of Hepatocellular Carcinom. Cancer Manag Res (2020) 12:9087–96. 10.2147/CMAR.S259243 PMC7532044 33061603

[76] Zhang B Li F Zhu Z Ding A Luo J. CircRNA CDR1as/miR-1287/Raf1 Axis Modulates Hepatocellular Carcinoma Progression Through MEK/ERK Pathwa. Cancer Manag Res (2020) 12:8951–64. 10.2147/CMAR.S252679 PMC7522432 33061591

[77] Liu Y Guo J Shen K Wang R Chen C Liao Z. Paclitaxel Suppresses Hepatocellular Carcinoma Tumorigenesis Through Regulating Circ-BIRC6/miR-877-5p/YWHAZ Axi. Onco Targets Ther (2020) 13:9377–88. 10.2147/OTT.S261700 PMC7519811 33061425

[78] Ji C Hong X Lan B Lin Y He Y Chen J. Circ_0091581 Promotes the Progression of Hepatocellular Carcinoma Through Targeting miR-591/FOSL2 Axi. Dig Dis Sci (2020). 10.1007/s10620-020-06641-4 33040214

[79] Zhao Z Song J Tang B Fang S Zhang D Zheng L. CircSOD2 Induced Epigenetic Alteration Drives Hepatocellular Carcinoma Progression Through Activating JAK2/STAT3 Signaling Pathway. J Exp Clin Cancer Res (2020) 39(1):259. 10.1186/s13046-020-01769-7 33234142 PMC7687771

[80] Yu YX Ge TW Zhang P. Circular RNA Circgfra1 Promotes Angiogenesis, Cell Proliferation and Migration of Hepatocellular Carcinoma by Combining With miR-149. Eur Rev Med Pharmacol Sci (2020) 24(21):11058–64. 10.26355/eurrev_202011_23591 33215421

[81] Zhang X Hao HH Zhuang HW Wang J Sheng Y Xu F. Circular RNA Circ_0008305 Aggravates Hepatocellular Carcinoma Growth Through Binding to miR-186 and Inducing TMED2. J Cell Mol Med (2020). 10.1111/jcmm.15945 PMC8918415 33210454

[82] Wei H Yan S Hui Y Liu Y Guo H Li Q. CircFAT1 Promotes Hepatocellular Carcinoma Progression *via* miR-30a-5p/REEP3 Pathway. J Cell Mol Med (2020) 24(24):14561–70. 10.1111/jcmm.16085 PMC7754024 33179443

[83] Zhu Z Shen S Zhao S Wang Z. Hsa_circ_0006916 Knockdown Represses the Development of Hepatocellular Carcinoma *via* Modulating miR-599/SRSF2 Axi. Onco Targets Ther (2020) 13:11301–13. 10.2147/OTT.S267471 PMC7649248 33177838

[84] Liu Q Wang C Jiang Z Li S Li F Tan HB. circRNA 001306 Enhances Hepatocellular Carcinoma Growth by Up-Regulating CDK16 Expression *via* Sponging miR-584-5p. J Cell Mol Med (2020) 24(24):14306–15. 10.1111/jcmm.16047 PMC7754030 33135290

[85] Yang TB Yi F Liu WF Yang YH Yang C Sun J. Identification of Hsa_Circ_0039053 as an Up-Regulated and Oncogenic circRNA in Hepatocellular Carcinoma *via* the miR-637-Mediated USP21 Activation. J Cancer (2020) 11(23):6950–9. 10.7150/jca.48998 PMC7591988 33123285

[86] Jiang Z Zhang Y Liu X Liang J Qiu G Zhu X. Identification of a Functional ceRNA Network to Explore Potential Biomarkers for Hepatocellular Carcinom. Onco Targets Ther (2020) 13:12341–55. 10.2147/OTT.S278912 PMC7719347 33293827

[87] Zhang Y Wang D Zhu T Yu J Wu X Lin W. CircPUM1 Promotes Hepatocellular Carcinoma Progression Through the miR-1208/MAP3K2 Axis. J Cell Mol Med (2021) 25(1):600–12. 10.1111/jcmm.15998 PMC7810943 33320435

[88] Guan Y Zhang Y Hao L Nie Z. CircRNA_102272 Promotes Cisplatin-Resistance in Hepatocellular Carcinoma by Decreasing MiR-326 Targeting of RUNX2. Cancer Manag Res (2020) 12:12527–34. 10.2147/CMAR.S258230 PMC7732977 33324096

[89] Chen CH Su YJ Ding H Duan J Wang J. Circular RNA ZNF292 Affects Proliferation and Apoptosis of Hepatocellular Carcinoma Cells by Regulating Wnt/beta-Catenin Pathway. Eur Rev Med Pharmacol Sci (2020) 24(23):12124–30. 10.26355/eurrev_202012_24001 33336730

[90] Li X He J Ren X Zhao H. Circ_0003998 Enhances Doxorubicin Resistance in Hepatocellular Carcinoma by Regulating miR-218-5p/EIF5A2 Pathway. Diagn Pathol (2020) 15(1):141. 10.1186/s13000-020-01056-1 33308276 PMC7733254

[91] Lv S Li Y Ning H Zhang M Jia Q Wang X. CircRNA GFRA1 Promotes Hepatocellular Carcinoma Progression by Modulating the miR-498/NAP1L3 Axis. Sci Rep (2021) 11(1):386. 10.1038/s41598-020-79321-y 33431945 PMC7801409

[92] Li L He K Chen S Wei W Tian Z Tang Y. Circ_0001175 Promotes Hepatocellular Carcinoma Cell Proliferation and Metastasis by Regulating miR-130a-5p. Onco Targets Ther (2020) 13:13315–27. 10.2147/OTT.S262408 PMC7781360 33408482

[93] Lyu P Zhai Z Hao Z Zhang H He J. CircWHSC1 Serves as an Oncogene to Promote Hepatocellular Carcinoma Progression. Eur J Clin Invest (2021) 51(6):e13487. 10.1111/eci.13487 33410156

[94] Yu Y Bian L Liu R Wang Y Xiao X. Circular RNA Hsa_Circ_0061395 Accelerates Hepatocellular Carcinoma Progression *via* Regulation of the miR-877-5p/PIK3R3 Axis. Cancer Cell Int (2021) 21(1):10. 10.1186/s12935-020-01695-w 33407443 PMC7788978

[95] Zhou D Dong L Yang L Ma Q Liu F Li Y. Identification and Analysis of circRNA-miRNA-mRNA Regulatory Network in Hepatocellular Carcinoma. IET Syst Biol (2020) 14(6):391–8. 10.1049/iet-syb.2020.0061 PMC8687197 33399102

[96] Xu R Yin S Zheng M Pei X Ji X. Circular RNA circZFR Promotes Hepatocellular Carcinoma Progression by Regulating miR-375/HMGA2 Axi. Dig Dis Sci (2021). 10.1007/s10620-020-06805-2 33433801

[97] Wen Z Guo YX Sun HD. Circular RNA Circsox4 Promotes the Proliferation, Migration and Apoptosis of Hepatocellular Carcinoma Cells by Down Regulating microRNA-432 Expression. Eur Rev Med Pharmacol Sci (2021) 25(4):1845–52. 10.26355/eurrev_202102_25079 33660794

[98] Weng H Zeng L Cao L Chen T Li Y Xu Y. Circfoxm1 Contributes to Sorafenib Resistance of Hepatocellular Carcinoma Cells by Regulating MECP2 *via* miR-1324. Mol Ther Nucleic Acids (2021) 23:811–20. 10.1016/j.omtn.2020.12.019 PMC7868711 33614231

[99] Si H Wang H Xiao H Fang Y Wu Z. Anti-Tumor Effect of Celastrol on Hepatocellular Carcinoma by the Circ_SLIT3/miR-223-3p/CXCR4 Axi. Cancer Manag Res (2021) 13:1099–111. 10.2147/CMAR.S278023 PMC7872924 33574707

[100] Fan W Chen L Wu X Zhang T. Circ_0031242 Silencing Mitigates the Progression and Drug Resistance in DDP-Resistant Hepatoma Cells by the miR-924/POU3F2 Axi. Cancer Manag Res (2021) 13:743–55. 10.2147/CMAR.S272851 PMC7847388 33531841

[101] Bu N Dong Z Zhang L Zhu W Wei F Zheng S. CircPVT1 Regulates Cell Proliferation, Apoptosis and Glycolysis in Hepatocellular Carcinoma *via* miR-377/TRIM23 Axi. Cancer Manag Res (2020) 12:12945–56. 10.2147/CMAR.S280478 PMC7751302 33364841

[102] Zhu C Su Y Liu L Wang S Liu Y Wu J. Circular RNA Hsa_Circ_0004277 Stimulates Malignant Phenotype of Hepatocellular Carcinoma and Epithelial-Mesenchymal Transition of Peripheral Cell. Front Cell Dev Biol (2020) 8:585565. 10.3389/fcell.2020.585565 33511111 PMC7835424

[103] Luo Z Lu L Tang Q Wei W Chen P Chen Y. CircCAMSAP1 Promotes Hepatocellular Carcinoma Progression Through miR-1294/GRAMD1A Pathway. J Cell Mol Med (2021) 25(8):3793–802. 10.1111/jcmm.16254 PMC8051675 33484498

[104] Wang L Zhou L Hou J Meng J Lin K Wu X. Three Novel circRNAs Upregulated in Tissue and Plasma From Hepatocellular Carcinoma Patients and Their Regulatory Network. Cancer Cell Int (2021) 21(1):72. 10.1186/s12935-021-01762-w 33482819 PMC7824949

[105] Yan F Fan B Wang J Wei W Tang Q Lu L. Circ_0008305-Mediated miR-660/BAG5 Axis Contributes to Hepatocellular Carcinoma Tumorigenesis. Cancer Med (2021) 10(3):833–42. 10.1002/cam4.3657 PMC7897943 33481351

[106] Jia Y Li S Zhang M Zhang Z Wang C Zhang C. Circ_LDLR Knockdown Suppresses Progression of Hepatocellular Carcinoma *via* Modulating miR-7/RNF38 Axi. Cancer Manag Res (2021) 13:337–49. 10.2147/CMAR.S275003 PMC7813465 33469375

[107] Li Y Zhang Y Zhang S Huang D Li B Liang G. circRNA Circarnt2 Suppressed the Sensitivity of Hepatocellular Carcinoma Cells to Cisplatin by Targeting the miR-155-5p/PDK1 Axi. Mol Ther Nucleic Acids (2021) 23:244–54. 10.1016/j.omtn.2020.08.037 PMC7772525 33425483

[108] Zhu Q Lu G Luo Z Gui F Wu J Zhang D. CircRNA Circ_0067934 Promotes Tumor Growth and Metastasis in Hepatocellular Carcinoma Through Regulation of miR-1324/FZD5/Wnt/beta-Catenin Axis. Biochem Biophys Res Commun (2018) 497(2):626–32. 10.1016/j.bbrc.2018.02.119 29458020

[109] Gao J Li E Liu W Yang Q Xie C Ai J. Circular RNA MYLK Promotes Hepatocellular Carcinoma Progression Through the Mir29a/KMT5C Signaling Pathwa. Onco Targets Ther (2020) 13:8615–27. 10.2147/OTT.S258715 PMC7457592 32904604

[110] Li Z Hu Y Zeng Q Wang H Yan J Li H. Circular RNA MYLK Promotes Hepatocellular Carcinoma Progression by Increasing Rab23 Expression by Sponging miR-362-3p. Cancer Cell Int (2019) 19:211. 10.1186/s12935-019-0926-7 31413665 PMC6688277

[111] He S Yang J Jiang S Li Y Han X. Circular RNA Circ_0000517 Regulates Hepatocellular Carcinoma Development *via* miR-326/IGF1R Axis. Cancer Cell Int (2020) 20:404. 10.1186/s12935-020-01496-1 32863763 PMC7448484

[112] He S Guo Z Kang Q Wang X Han X. Circular RNA Hsa_Circ_0000517 Modulates Hepatocellular Carcinoma Advancement *via* the miR-326/SMAD6 Axis. Cancer Cell Int (2020) 20:360. 10.1186/s12935-020-01447-w 32774154 PMC7397604

[113] Zang H Li Y Zhang X Huang G. Circ_0000517 Contributes to Hepatocellular Carcinoma Progression by Upregulating TXNDC5 *via* Sponging miR-1296-5p. Cancer Manag Res (2020) 12:3457–68. 10.2147/CMAR.S244024 PMC7234960 32523376

[114] Li Y Shi H Yuan J Qiao L Dong L Wang Y. Downregulation of Circular RNA Circpvt1 Restricts Cell Growth of Hepatocellular Carcinoma Through Downregulation of Sirtuin 7 *via* microRNA-3666. Clin Exp Pharmacol Physiol (2020) 47(7):1291–300. 10.1111/1440-1681.13273 32017171

[115] Zhu Y Liu Y Xiao B Cai H Liu M Ma L. The Circular RNA PVT1/miR-203/HOXD3 Pathway Promotes the Progression of Human Hepatocellular Carcinoma. Biol Open (2019) 8(9):bio043687. 10.1242/bio.043687 31551242 PMC6777361

[116] Liu L Yang X Li NF Lin L Luo H. Circ_0015756 Promotes Proliferation, Invasion and Migration by microRNA-7-Dependent Inhibition of FAK in Hepatocellular Carcinoma. Cell Cycle (2019) 18(21):2939–53. 10.1080/15384101.2019.1664223 PMC6791692 31522588

[117] Guo W Zhao L Wei G Liu P Zhang Y Fu L. Circ_0015756 Aggravates Hepatocellular Carcinoma Development by Regulating FGFR1 *via* Sponging miR-610. Cancer Manag Res (2020) 12:7383–94. 10.2147/CMAR.S262231 PMC7443463 32884351

[118] Wang M Yang Y Yang J Han S. Circ_KIAA1429 Accelerates Hepatocellular Carcinoma Advancement Through the Mechanism of M(6)A-YTHDF3-Zeb1. Life Sci (2020) 257:118082. S0024-3205(20)30833-X 32653519 10.1016/j.lfs.2020.118082

[119] Weng Q Chen M Li M Zheng YF Shao G Fan W. Global Microarray Profiling Identified Hsa_Circ_0064428 as a Potential Immune-Associated Prognosis Biomarker for Hepatocellular Carcinoma. J Med Genet (2019) 56(1):32–8. 10.1136/jmedgenet-2018-105440 30120213

[120] Liang WC Wong CW Liang PP Shi M Cao Y Rao ST. Translation of the Circular RNA Circbeta-Catenin Promotes Liver Cancer Cell Growth Through Activation of the Wnt Pathway. Genome Biol (2019) 20(1):84. 10.1186/s13059-019-1685-4 31027518 PMC6486691

[121] Wang L Long H Zheng Q Bo X Xiao X Li B. Circular RNA Circrhot1 Promotes Hepatocellular Carcinoma Progression by Initiation of NR2F6 Expression. Mol Cancer (2019) 18(1):119. 10.1186/s12943-019-1046-7 31324186 PMC6639939

[122] Cheng F Wang L Zhang J. Circular RNA 0016788 Displays as a Biomarker for Tumor Progression and Poor Prognosis in Surgical Hepatocellular Carcinoma Patients. J Clin Lab Anal (2020) 34(7):e23300. 10.1002/jcla.23300 32319701 PMC7370714

[123] Qiao GL Chen L Jiang WH Yang C Yang CM Song LN. Hsa_circ_0003998 may be Used as a New Biomarker for the Diagnosis and Prognosis of Hepatocellular Carcinoma. Onco Targets Ther (2019) 12:5849–60. 10.2147/OTT.S210363 PMC6650091 31410028

[124] Zhang X Xu Y Qian Z Zheng W Wu Q Chen Y. circRNA_104075 Stimulates YAP-Dependent Tumorigenesis Through the Regulation of HNF4a and May Serve as a Diagnostic Marker in Hepatocellular Carcinoma. Cell Death Dis (2018) 9(11):1091. 10.1038/s41419-018-1132-6 30361504 PMC6202383

[125] Wang W Li Y Li X Liu B Han S Zhang B. Circular RNA Circ-FOXP1 Induced by SOX9 Promotes Hepatocellular Carcinoma Progression *via* Sponging miR-875-3p and miR-421. BioMed Pharmacother (2020) 121:109517. S0753-3322(19)34002-8 31698267 10.1016/j.biopha.2019.109517

[126] Sun XH Wang YT Li GF Zhang N Fan L. Serum-Derived three-circRNA Signature as a Diagnostic Biomarker for Hepatocellular Carcinoma. Cancer Cell Int (2020) 20:226. 10.1186/s12935-020-01302-y 32536814 PMC7288432

[127] Lei B Zhou J Xuan X Tian Z Zhang M Gao W. Circular RNA Expression Profiles of Peripheral Blood Mononuclear Cells in Hepatocellular Carcinoma Patients by Sequence Analysis. Cancer Med (2019) 8(4):1423–33. 10.1002/cam4.2010 PMC6488130 30714679

[128] Li Y Zang H Zhang X Huang G. Exosomal Circ-ZNF652 Promotes Cell Proliferation, Migration, Invasion and Glycolysis in Hepatocellular Carcinoma *via* miR-29a-3p/GUCD1 Axi. Cancer Manag Res (2020) 12:7739–51. 10.2147/CMAR.S259424 PMC7473989 32943922

[129] Lai Z Wei T Li Q Wang X Zhang Y Zhang S. Exosomal Circfblim1 Promotes Hepatocellular Carcinoma Progression and Glycolysis by Regulating the miR-338/LRP6 Axi. Cancer Biother Radiopharm (2020). 10.1089/cbr.2020.3564 32907351

[130] Zhou S Wei J Wang Y Liu X. Cisplatin Resistance-Associated circRNA_101237 Serves as a Prognostic Biomarker in Hepatocellular Carcinoma. Exp Ther Med (2020) 19(4):2733–40. 10.3892/etm.2020.8526 PMC7092923 32226487

[131] Yang G Wang X Liu B Lu Z Xu Z Xiu P. Circ-BIRC6, a Circular RNA, Promotes Hepatocellular Carcinoma Progression by Targeting the miR-3918/Bcl2 Axis. Cell Cycle (2019) 18(9):976–89. 10.1080/15384101.2019.1601477 PMC6527301 30931701

[132] Luo Y Liu F Gui R. High Expression of Circulating Exosomal Circakt3 Is Associated With Higher Recurrence in HCC Patients Undergoing Surgical Treatment. Surg Oncol (2020) 33:276–81. S0960-7404(19)30601-2 10.1016/j.suronc.2020.04.021 32561093

[133] Qin M Liu G Huo X Tao X Sun X Ge Z. Hsa_circ_0001649: A Circular RNA and Potential Novel Biomarker for Hepatocellular Carcinoma. Cancer Biomark (2016) 16(1):161–9. 10.3233/CBM-150552 PMC13016540 26600397

[134] Yao Z Luo J Hu K Lin J Huang H Wang Q. ZKSCAN1 Gene and Its Related Circular RNA (Circzkscan1) Both Inhibit Hepatocellular Carcinoma Cell Growth, Migration, and Invasion But Through Different Signaling Pathways. Mol Oncol (2017) 11(4):422–37. 10.1002/1878-0261.12045 PMC5527481 28211215

[135] Zhang Y Wang H Li C Gao L Zheng Y Chang W. CircSMYD4 Regulates Proliferation, Migration and Apoptosis of Hepatocellular Carcinoma Cells by Sponging miR-584-5p. Cancer Cell Int (2020) 20(1):556. 10.1186/s12935-020-01648-3 33292243 PMC7678128

[136] Chen H Liu S Li M Huang P Li X. Circ_0003418 Inhibits Tumorigenesis And Cisplatin Chemoresistance Through Wnt/beta-Catenin Pathway In Hepatocellular Carcinom. Onco Targets Ther (2019) 12:9539–49. 10.2147/OTT.S229507 PMC6857737 31807029

[137] Shi M Li ZY Zhang LM Wu XY Xiang SH Wang YG. Hsa_circ_0007456 Regulates the Natural Killer Cell-Mediated Cytotoxicity Toward Hepatocellular Carcinoma *via* the miR-6852-3p/ICAM-1 Axis. Cell Death Dis (2021) 12(1):94. 10.1038/s41419-020-03334-8 33462208 PMC7814008

[138] Fu L Yao T Chen Q Mo X Hu Y Guo J. Screening Differential Circular RNA Expression Profiles Reveals Hsa_Circ_0004018 Is Associated With Hepatocellular Carcinoma. Oncotarget (2017) 8(35):58405–16. 10.18632/oncotarget.16881 PMC5601662 28938566

[139] Fu L Wu S Yao T Chen Q Xie Y Ying S. Decreased Expression of Hsa_Circ_0003570 in Hepatocellular Carcinoma and Its Clinical Significance. J Clin Lab Anal (2018) 32(2):e22239. 10.1002/jcla.22239 PMC6816847 28493512

[140] Shi L Yan P Liang Y Sun Y Shen J Zhou S. Circular RNA Expression Is Suppressed by Androgen Receptor (AR)-Regulated Adenosine Deaminase That Acts on RNA (ADAR1) In Human Hepatocellular Carcinoma. Cell Death Dis (2017) 8(11):e3171. 10.1038/cddis.2017.556 29144509 PMC5775411

[141] Yu J Xu QG Wang ZG Yang Y Zhang L Ma JZ. Circular RNA Csmarca5 Inhibits Growth and Metastasis in Hepatocellular Carcinoma. J Hepatol (2018) 68(6):1214–27. S0168-8278(18)30055-2[ 10.1016/j.jhep.2018.01.012 29378234

[142] Guo W Zhang J Zhang D Cao S Li G Zhang S. Polymorphisms and Expression Pattern of Circular RNA Circ-ITCH Contributes to the Carcinogenesis of Hepatocellular Carcinoma. Oncotarget (2017) 8(29):48169–77. 10.18632/oncotarget.18327 PMC5564635 28636993

[143] Yang W Ju HY Tian XF. Circular RNA-ABCB10 Suppresses Hepatocellular Carcinoma Progression Through Upregulating NRP1/ABL2 *via* Sponging miR-340-5p/miR-452-5p. Eur Rev Med Pharmacol Sci (2020) 24(5):2347–57. 10.26355/eurrev_202003_20501 32196586

[144] Li R Deng Y Liang J Hu Z Li X Liu H. Circular RNA Circ-102,166 Acts as a Sponge of miR-182 and miR-184 to Suppress Hepatocellular Carcinoma Proliferation and Invasion. Cell Oncol (Dordr) (2020) 44(2):279–95. 10.1007/s13402-020-00564-y PMC12980780 33034848

[145] Wu M Sun T Xing L. Circ_0004913 Inhibits Cell Growth, Metastasis, and Glycolysis by Absorbing miR-184 to Regulate HAMP in Hepatocellular Carcinom. Cancer Biother Radiopharm (2020). 10.1089/cbr.2020.3779 33021399

[146] Zhu P Liang H Huang X Zeng Q Liu Y Lv J. Circular RNA Hsa_circ_0004018 Inhibits Wnt/beta-Catenin Signaling Pathway by Targeting microRNA-626/DKK3 in Hepatocellular Carcinom. Onco Targets Ther (2020) 13:9351–64. 10.2147/OTT.S254997 PMC7519839 33061423

[147] Luo Y Fu Y Huang R Gao M Liu F Gui R. CircRNA_101505 Sensitizes Hepatocellular Carcinoma Cells to Cisplatin by Sponging miR-103 and Promotes Oxidored-Nitro Domain-Containing Protein 1 Expression. Cell Death Discov (2019) 5:121. 10.1038/s41420-019-0202-6 31372241 PMC6662675

[148] Liu H Lan T Li H Xu L Chen X Liao H. Circular RNA Circdlc1 Inhibits MMP1-Mediated Liver Cancer Progression *via* Interaction With Hu. Theranostics (2021) 11(3):1396–411. 10.7150/thno.53227 PMC7738888 33391541

[149] Ma H Huang C Huang Q Li G Li J Huang B. Circular RNA Circ_0014717 Suppresses Hepatocellular Carcinoma Tumorigenesis Through Regulating miR-668-3p/BTG2 Axi. Front Oncol (2020) 10:592884. 10.3389/fonc.2020.592884 33598424 PMC7883829

[150] Gu Y Wu F Wang H Chang J Wang Y Li X. Circular RNA Circarpp21 Acts as a Sponge of miR-543 to Suppress Hepatocellular Carcinoma by Regulating LIF. Onco Targets Ther (2021) 14:879–90. 10.2147/OTT.S283026 PMC7874301 33584097

[151] Bu FT Zhu Y Chen X Wang A Zhang YF You HM. Circular RNA Circpsd3 Alleviates Hepatic Fibrogenesis by Regulating the miR-92b-3p/Smad7 Axis. Mol Ther Nucleic Acids (2021) 23:847–62. 10.1016/j.omtn.2021.01.007 PMC7868733 33614234

[152] Zhong L Wang Y Cheng Y Wang W Lu B Zhu L. Circular RNA Circc3p1 Suppresses Hepatocellular Carcinoma Growth and Metastasis Through miR-4641/PCK1 Pathway. Biochem Biophys Res Commun (2018) 499(4):1044–9. S0006-291X(18)30754-X 10.1016/j.bbrc.2018.03.221 29608893

[153] Han D Li J Wang H Su X Hou J Gu Y. Circular RNA Circmto1 Acts as the Sponge of microRNA-9 to Suppress Hepatocellular Carcinoma Progression. Hepatology (2017) 66(4):1151–64. 10.1002/hep.29270 28520103

[154] Fu L Chen Q Yao T Li T Ying S Hu Y. Hsa_circ_0005986 Inhibits Carcinogenesis by Acting as a miR-129-5p Sponge and Is Used as a Novel Biomarker for Hepatocellular Carcinoma. Oncotarget (2017) 8(27):43878–88. 10.18632/oncotarget.16709 PMC5546447 28410211

[155] Chen W Quan Y Fan S Wang H Liang J Huang L. Exosome-Transmitted Circular RNA Hsa_Circ_0051443 Suppresses Hepatocellular Carcinoma Progression. Cancer Lett (2020) 475:119–28. S0304-3835(20)30036-7 10.1016/j.canlet.2020.01.022 32014458

[156] Li Z Zhou Y Yang G He S Qiu X Zhang L. Using Circular RNA SMARCA5 as a Potential Novel Biomarker for Hepatocellular Carcinoma. Clin Chim Acta (2019) 492:37–44. S0009-8981(19)30053-1 30716279 10.1016/j.cca.2019.02.001

[157] Avilala J Becnel D Abdelghani R Nanbo A Kahn J Li L. Role of Virally Encoded Circular RNAs in the Pathogenicity of Human Oncogenic Viruse. Front Microbiol (2021) 12:657036. 10.3389/fmicb.2021.657036 33959113 PMC8093803

[158] Zhu M Liang Z Pan J Hu X Zhang X Xue R. HBV pgRNA Can Generate A CircRNA with Two Junction Sites. bioRxiv [Preprint] (2020) 38(5). 10.1101/2020.05.14.095273

[159] Farooqi AA Naureen H Attar R. Regulation of Cell Signaling Pathways by Circular RNAs and microRNAs in Different Cancers: Spotlight on Wnt/beta-Catenin, JAK/STAT, TGF/SMAD, SHH/GLI, NOTCH and Hippo Pathways. Semin Cell Dev Biol (2021). S1084-9521(21)00075-6 10.1016/j.semcdb.2021.04.002 33863643

[160] Zhang Q Xu M Qu Y Li Z Cai X Lu L. Analysis of the Differential Expression of Circulating microRNAs During the Progression of Hepatic Fibrosis in Patients With Chronic Hepatitis B Virus Infection. Mol Med Rep (2015) 12(4):5647–54. 10.3892/mmr.2015.4221 PMC4581744 26299203

[161] Han N Ding L Wei X Fan L Yu L. Circsmad2 Governs Migration and Epithelial-Mesenchymal Transition by Inhibiting microRNA-9. J Cell Biochem (2019). 10.1002/jcb.29638 31886568

[162] Zhang X Luo P Jing W Zhou H Liang C Tu J. Circsmad2 Inhibits the Epithelial-Mesenchymal Transition by Targeting miR-629 in Hepatocellular Carcinoma. Onco Targets Ther (2018) 11:2853–63. 10.2147/OTT.S158008 PMC5962255 29844683

[163] Wang Z Kawaguchi K Honda M Hashimoto S Shirasaki T Okada H. Notch Signaling Facilitates Hepatitis B Virus Covalently Closed Circular DNA Transcription *via* cAMP Response Element-Binding Protein With E3 Ubiquitin Ligase-Modulation. Sci Rep (2019) 9(1):1621. 10.1038/s41598-018-38139-5 30733490 PMC6367350

[164] Zhao J Qi YF Yu YR. STAT3: A Key Regulator in Liver Fibrosis. Ann Hepatol (2021) 21:100224. S1665-2681(20)30071-5 32702499 10.1016/j.aohep.2020.06.010

[165] Sun S Gao J Zhou S Li Y Wang Y Jin L. A Novel Circular RNA Circ-LRIG3 Facilitates the Malignant Progression of Hepatocellular Carcinoma by Modulating the EZH2/STAT3 Signaling. J Exp Clin Cancer Res (2020) 39(1):252. 10.1186/s13046-020-01779-5 33222697 PMC7682056

[166] Yang L Xue H Sun Y Zhang L Xue F Ge R. CircularRNA-9119 Protects Hepatocellular Carcinoma Cells From Apoptosis by Intercepting miR-26a/JAK1/STAT3 Signaling. Cell Death Dis (2020) 11(7):605. 10.1038/s41419-020-02807-0 32732872 PMC7393165

[167] Timperi E Barnaba V. Viral Hepatitides, Inflammation and Tumour Microenvironmen. Adv Exp Med Biol (2020) 1263:25–43. 10.1007/978-3-030-44518-8_3 32588321

[168] Zhou J Yu L Gao X Hu J Wang J Dai Z. Plasma microRNA Panel to Diagnose Hepatitis B Virus-Related Hepatocellular Carcinoma. J Clin Oncol (2011) 29(36):4781–8. JCO.2011.38.2697 10.1200/JCO.2011.38.2697 22105822

[169] Huang XY Huang ZL Zhang PB Huang J Wang HC Xu B. CircRNA-100338 Is Associated With mTOR Signaling Pathway and Poor Prognosis in Hepatocellular Carcinom. Front Oncol (2019) 9:392. 10.3389/fonc.2019.00392s 31157168 PMC6528706

[170] Ding B Lou W Xu L Fan W. Non-Coding RNA in Drug Resistance of Hepatocellular Carcinoma. Biosci Rep (2018) 38(5):BSR20180915. 10.1042/BSR20180915 30224380 PMC6177555

